# High effective time-dependent THz spectroscopy method for the detection and identification of substances with inhomogeneous surface

**DOI:** 10.1371/journal.pone.0201297

**Published:** 2018-08-09

**Authors:** Vyacheslav A. Trofimov, Svetlana A. Varentsova

**Affiliations:** Faculty of Computational Mathematics and Cybernetics, Lomonosov Moscow State University, Moscow, Russia; Queensland University of Technology, AUSTRALIA

## Abstract

We discuss an effective time-dependent THz spectroscopy method for the detection and identification of a substance with an inhomogeneous surface using a broadband THz signal reflected from the substance. We show that a successful and reliable identification can be made using the single long-duration THz signal, which contains not only the main reflected pulse, but also several sub-pulses. The method does not use averaging of the measured THz signals over the viewing angles and scanning over the surface area, which significantly increases the signal processing speed. The identification is based on the method of spectral dynamics analysis together with the integral correlation criteria (ICC). We compare the absorption spectral dynamics of a substance under analysis with the corresponding dynamics for a standard substance from database. For reliable and effective substance detection, we propose to use several ICC simultaneously in different time intervals, which contain not only the main pulse of the reflected THz signal, but also the sub-pulses. This way, one can detect and identify the substance in the sample with high probability. As examples of identification, we used the THz signals reflected from the plastic explosive PWM C4 with both rough and concave surface. We show that the main pulse, reflected from the inhomogeneous surface of the sample, contains information about its absorption frequencies.

## 1. Introduction

Currently, active research is being carried out worldwide to develop methods for applying THz radiation for solving security and anti-terrorism problems. This is connected primarily with the fact that THz radiation is non-ionizing, safe for human and animals exposure at low power levels. Furthermore, the most common non-polar substances are transparent to it and have spectral fingerprints in the THz frequency range [1–12]. These factors allow the use of THz spectrometers not only for security screening problems but for many research in scientific and applied fields including non-destructive testing [13], [14], investigation of paintings on canvas [15], quality control inspection of pharmaceuticals and food products [16], [17] as well as for medical and biological applications [18].

In most THz TDS setups, the substance identification occurs based on comparison of the absorption frequencies of a substance under investigation with a set of known absorption frequencies from a database. Below, we call this identification technique as the standard THz TDS method. Despite the potential benefits of this technology, it has well-known disadvantages. If the object is masked by some non-opaque covering or hidden under the packaging material, then the spectrum of the measured THz signal can be significantly attenuated and distorted [6]-[9]. The influence of water vapor also limits the ability of THz TDS for remote measurements, in particular due to the strong intensity variation of the water vapor absorption lines when the air humidity changes [10].

One more important task, which needs to be solved before the creation of security screening devices, which can effectively operate under real conditions, is the influence of the inhomogeneity of the substance surface on the spectral characteristics of this substance. It is well known that surface roughness or/and curvature can lead to scattering and modulating of the spectrum, which complicates the identification [19]-[26] of the test object. In [19] the scattering was modeled due to granularity of materials using the quasi-crystalline approximation and it was shown that such scattering results in a decrease of the signal bandwidth after the THz waves transmit through the sample pellets. In [20] an analytical model is presented that shows that reflection from a rough surface causes a Gaussian frequency roll-off for the spectral magnitude of a terahertz wave and reduces the signal-to-noise ratio of terahertz time-domain spectroscopy. In addition to the articles describing the scattering processes, in [21]-[25] methods were proposed for eliminating these effects. In [21] it was shown that by summing and averaging multiple measurements over a sample area (10x10 mm^2^, 2500 disjoint transmission measurements, step size 200 *μ*m, overall measurement time 2 min), the granularity scattering effect can be effectively decreased. As shown in [21], the reliable THz spectrum of granulated sucrose with a single absorption frequency with maximal spectral amplitude was obtained.

It is worth noting that in the reflection mode, surface roughness is the main source of terahertz scattering [21], [22]. In [22] the authors showed that a surface roughness, even smaller than the radiation wavelengths, may prevent the observation of most spectral signatures due to a high-frequency roll-off of the specular reflectance. However, on increasing the angle of incidence from 10° up to 60°, the roll-off occurred at higher frequency. The pellet with explosive HMX was used as a sample with a surface roughness parameter σ = 9.2 μm. However, such parameter σ corresponds to ultrafine sand paper, which is used for final sanding and polishing of thick materials (https://en.wikipedia.org/wiki/Sandpaper). Thus, this example is far from realistic surface roughness with 425 μm > σ > 115 μm (the corresponding sand paper grit varies from 40 to 120) and the results obtained cannot be used in real security screening systems.

In [23] the effects of rough surfaces on a spectroscopic signature using diffuse and specular returns from rough lactose were investigated. The samples were pressed with realistic 40, 80, and 120 grit sandpaper. In the specular case, it was shown that the rough surface lowers the strength of the absorption peak, obtained by taking the negative derivative of the reflection. Increasing the number of viewing angles (up to 17 realizations from -16° to 16°) can substantially help to distinguish the signature of the material from the random noise due to roughness. The first (and only) absorption peak of lactose near 0.54 THz was obtained.

In [24] the scattering from a lactose surface with the roughness parameter of 40 grit sandpaper was simulated using the Kirchhoff Approximation (KA) model. A total of 900 surface realizations were simulated, and the negative derivative of the scattered power with respect to frequency was calculated for 30 sets of 30 surfaces. The small peak at 0.54 THz is obtained due to averaging over multiple surfaces and diffuse angles. However, within each data set, the small peak at 0.54 THz is obscured by higher peaks which are due to random surface scattering. It is shown that a low-pass cepstrum filter can be used to reduce noise.

Thus, despite the fact that the spectrum of the signal under investigation contains several absorption frequencies, the authors in [21]-[24] can reliably identify only one absorption frequency with maximal spectral amplitude.

In [25] an implementation of wavelet methods that can retrieve spectroscopic information using only a few spatially disjoint reflection measurements was presented. However, in [25] it was also shown that “if two features are too close to each other in the frequency domain, separate identification of each line can be potentially challenging and will depend on the broadness of each feature and the frequency resolution of the measurement.”

It should be also noted that currently active work is underway to create THz metamaterials with optical properties that are absent in natural materials. Thus, in [26] using the 1-bit coding metamaterial, the authors successfully obtained a low-reflection and scattering surface with a wide THz frequency range (0.77−1.38 THz) and a wide range of incident angle (0°–40°).

Unlike the methods mentioned above, in the discussed method we use only one THz signal reflected from the substance with inhomogeneous surface but measured in the long time interval duration 180–200 ps. This duration allows registering not only the main reflected THz pulse, but also several sub-pulses following it, which are due to the reflectance from the inner surfaces of the sample. These sub-pulses also contain information about spectral characteristics of the substance and can be used for the identification. Also, we do not apply averaging of measurements over viewing angles and the sample area in order to reduce the scattering effects. From our point of view, there are several restrictions for this technique application under real conditions. First, in practice it is necessary to make the substance identification in real time. Therefore, a number of realizations cannot be significantly increased; 2 min for 1800 realizations [21] or 900 surface realizations [24] is too long for real-time security screening. Thus, averaging over several hundred realizations reduces the performance of the security screening. Secondly, portable security screening devices may have a limited set of functions, including limitations on the viewing angle of measurement. Therefore, under real conditions it may be impossible to make averaging on a sufficiently large number of viewing angles to reduce noise due to the random rough surface. Thirdly, averaging over the sample area and viewing angles allows us to recover the absorption frequencies with maximal spectral amplitude while those with less amplitude are visible significantly worse. This reduces the accuracy and reliability of the method.

In the present paper, we continue our investigation of the spectral properties of the plastic explosive PWM C4 with inhomogeneous (rough and concave) surface in THz frequency range in reflection mode. We show that in both cases, such a surface distorts the spectrum and reflectance of the main pulse of the measured THz signal in such a way that the standard THz TDS method is inefficient in this situation for substance detection and identification. In order to overcome the standard THz TDS method limitations, we propose to use the spectral dynamics analysis method (SDA-method) and integral correlation criteria (ICC) for the detection and identification of a substance with an inhomogeneous surface.

It should be noted that the main feature of the integral correlation criteria consists in summing the correlation coefficients between spectral intensity evolution at a chosen frequency for the THz signal under investigation and the standard evolution from database. The summation is made during the time intervals of the THz signal analysis, which contain the main pulse or the first sub-pulse, or the remote part of this signal. This procedure is similar to noise suppression in signal processing and allows us to decrease the influence of random fluctuation of correlation coefficient on a probability estimation of the presence of the standard substance absorption frequencies in the investigated THz signal. In addition, we show that the ICC’s allows the detection not only of the single substance absorption frequency with the maximum amplitude, but also those absorption frequencies with less amplitude, which are not visible with the methods of averaging [21]-[25].

Earlier in [27]-[29] we demonstrated the possibility of using the SDA-method for the detection and identification of various neutral and dangerous substances in the transmission and reflection mode at short (no more than 30 cm) distances. In [30]-[32] we proposed to use for this purpose the ICC together with the SDA-method. In [33–35], the spectral properties of THz pulses measured at long distances of about 3.5 meters and at non-zero humidity were investigated by means of the ICC. In [36], [37] the ICCs were applied for substance detection and identification using the high noise THz signal. The essential limitations of the standard THz-TDS method were demonstrated as well in [35]-[37]. In [38] a computer simulation of a THz pulse interaction with disordered layered structure was made and the occurrence of false absorption frequencies in the spectrum of the signal transmitted through or reflected from such structure was explained. The effectiveness of the method for the detection and identification of the substances based on application of the ICC was confirmed.

The current paper continues the investigations reported in [29], [35], [37]. In [35], [37] we used for the detection and identification of the plastic explosive PWM C4 one of the first variants of the ICC, which takes into account both the spectral brightness of the standard THz signal and that of the THz signal under investigation. In these papers, the identification was carried out in the time intervals, which do not contain the main pulse of the reflected THz signal. In the current paper, in order to increase the accuracy and reliability of identification, we apply for this purpose several ICC’s simultaneously. The first ICC uses only the spectral brightness of the standard THz signal. The second ICC estimates the integral correlation between the dynamics of the THz signal under investigation and that of the standard THz signal, and it does not use the weight coefficients at all. As additional information about the substance presence in the sample with PWM C4, we use the integral correlation between the measured THz signal, reflected from this sample, and the standard THz signals transmitted through the most common explosives and illicit drugs under laboratory conditions.

Compared with the use of a single criterion, the use of these three criteria simultaneously in different time intervals, which contain not only the main pulse of the reflected THz signal, but also the sub-pulses, increases the probability and reliability of substance detection and identification and reduces the false positive and negative alarms.

The detection and identification of different types of explosives and illicit drugs by means of the SDA-method together with various types of the ICC is described in a number of our previous papers. Thus, in [32] the SDA-method together with the first types of the ICC was applied for the detection and identification of explosives RDX, HMX, PETN and two mixtures of explosives with three components each in reflection mode. The approach discussed in the current paper was successfully applied in [36], [39] for the detection and identification of illicit drugs MA, MDA, MDMA in very noisy THz signals as well as for detection of explosive HMX absorption frequencies in the THz signal reflected from the pellet with a smooth surface.

It should be noted that reflected THz signals measurements for PWM C4 were made in the Military University of Technology (Warsaw, Poland). THz signals transmitted through the RDX, HMX, PETM and TNT samples were measured in the Center for Terahertz Research, Rensselaer Polytechnic Institute (NY, USA), transmitted THz signals MA, MDA, MDMA and Ketamine—at Capital Normal University (Beijing, China).

## 2. Integral correlation criteria and the SDA-method

The SDA-method is a continuation and development of the standard THz-TDS technique for the transmitted or reflected broadband THz signal. Usually, the standard THz TDS analyzes the signal spectrum, which is averaged over a time interval during the pulse action and does not take into account the changes of spectral intensities at the full time interval or on its parts. In contrast to it, in the SDA-method the object of research are the dynamics of the spectral intensities (i.e. their evolution in time) in the time intervals, which contain the main pulse or sub-pulse, or the remote part of the THz signal at chosen frequencies. Each spectral intensity dynamics (or spectral line dynamics) has its individual shape and provides unique information about both the spectral intensity time evolution and the relaxation times of excited energy levels. This allows us to obtain the unique 2D signature of the substance in the THz frequency range.

In [30], [31] we proposed to use for substance detection and identification the integral correlation between the spectral intensity dynamics of the THz signal under investigation *S*(*t*) (reflected or transmitted) and that of the standard transmitted signal *s*(*t*) from database. Note that the calculation of the spectral intensity dynamics is described in a number of our previous papers, for example, in [35], [37]. Below for the readers’ convenience, we repeat the main notations and expressions for the ICC’s that are used below in Section 3.

The set of absolute values of spectral intensities for the standard signal *s*(*t*) at the chosen frequency *ν*_1_ is denoted as $p_{\nu_{1}}=\left\{\left|p_{\nu_{1}}^{}(t_{m})\right|\right\}$, *m* = 1,…,*M*_1_. The corresponding set for the signal under investigation *S*(*t*) at the frequency *ν*_2_ is denoted as $P_{\nu_{2}}=\left\{\left|P_{\nu_{2}}^{}(t_{m})\right|\right\}$, *m* = 1,…,*M*_2_. Its part that begins at time moment *t*_*n*_ and contains also *M*_1_ components, is denoted as $P_{\nu_{2}}{}^{\left(n\right)}=\left\{\left|P_{\nu_{2}}^{\left(n\right)}(t_{n+m})\right|\right\}$. Here *M*_1_ and *M*_2_ are the numbers of time moments in the full dynamics, they depend on the dynamics construction parameters—the window length T and its shift Δ along the signal. Obviously, these parameters must coincide for both dynamics $p_{\nu_{1}}$ and $P_{\nu_{2}}$. In the current paper, as in our previous articles [32]-[38], these parameters were chosen as follows: the window length T = 2.8 ps, its shift Δ = 0.2 ps.

On order to get the correlation coefficient between the sets $p_{\nu_{1}}$ and $P_{\nu_{2}}$ in each time moment *t*_*n*_, we move the standard set $p_{\nu_{1}}$ along the set $P_{\nu_{2}}$ and use the well-known expression:

$$c_{p,P}(t_{n})=\sum_{m=0}^{M_{1}-1}\left(|p_{\nu_{1}}(t_{m})|-\bar{p_{\nu_{1}}})\cdot (|P_{\nu_{2}}(t_{m+n})|-\bar{P_{\nu_{2}}}\right)/\|p_{{}_{\nu_{1}}}-\bar{p_{\nu_{1}}}\|\cdot \|P_{\nu_{2}}^{\left(n\right)}-\bar{P_{\nu_{2}}}\|,$$ (1)

where $\bar{p_{\nu_{1}}}=\sum_{m=0}^{M_{1}-1}\left|p_{\nu_{1}}(t_{m})\right|/M_{1}$, $\bar{P_{\nu_{2}}}=\sum_{m=0}^{M_{1}-1}\left|P_{\nu_{2}}(t_{m+n})\right|/M_{1}$.

In Section 3, for substance detection and identification we use the ICC’s *C*_*p*,*P*_(*t*_*n*_), *CW*_*p*,*P*_(*t*_*n*_) and *CW*1_*p*,*P*_(*t*_*n*_), which are based on correlation coefficient (1):

$$C_{p,P}^{}(t_{n})=\sum_{m=0}^{n}\left|c_{p,P}(t_{m})\right|$$ (2)

The ICC *CW*_*p*,*P*_(*t*_*n*_) takes into account the spectral intensity at each of frequencies ν_1_ and ν_2_ for both the signal under investigation and the standard signal during the time interval of correlation:

$$CW_{p,P}^{}(t_{n})=\sum_{m=0}^{n}\left|c_{p,P}(t_{m})\right|w_{1}w_{2},\;n=0,\ldots ,M_{2}-M_{1},$$ (3)

where *w*_1_ = *w*(|*P*(*ν*_1_)|), *w*_2_ = *w*(|*P*(*ν*_2_)|) are the weight coefficients. When *w*_1_ = 1, *w*_2_ = 1, we get the ICC *C*_*p*,*P*_(*t*_*n*_) (2).

In the current paper, we also use the ICC *CW*1_*p*,*P*_(*t*_*n*_), in which the weight coefficients are chosen in the following way: *w*_1_ = 1/|*P*(*ν*_1_)|, *w*_2_ = 1:

$$CW1_{p,P}^{}(t_{n})=\sum_{m=0}^{n}\left|c_{p,P}(t_{m})\right|w_{1},\;n=0,\ldots ,M_{2}-M_{1}.$$ (4)

In comparison with the ICC *CW*_*p*,*P*_(*t*_*n*_) (3), the ICC *CW*1_*p*,*P*_(*t*_*n*_) (4) uses only the spectral brightness of the standard signal *s*(*t*), not the signal under investigation *S*(*t*). This allows us to be less dependent on random fluctuation influence and to rely upon the spectral characteristics of the standard signal.

In [35]-[37], we introduced the following definition at using the ICC. The frequency ν is detected in the signal under investigation, if the corresponding ICC calculated for the pair (ν, ν_1_) lies above all other ICC in the frequency detection range (FDR). Here the frequency ν_1_ belongs to a standard signal spectrum. Vice versa, the frequency ν is not detected if there is at least one of other ICC lies above the ICC corresponding to this pair in this frequency range. As boundaries of the FDR, earlier in [35]-[37] we used the spectrum maxima closest to the analyzed frequency. Below we show that the boundaries of FDR can be defined in another way and for different integral criteria, the FDR’s may be different.

## 3. Identification of PWM C4 explosive with inhomogeneous surface in reflection mode

In the present section, we apply the integral correlation criteria (1)-(4) for the detection and identification of the dangerous substance with inhomogeneous surface. As an example of such a substance, we use the pellet with plastic explosive PWM C4, which is the mixture of explosive RDX and plasticizer in the ratio 9:1. In order to increase the accuracy and reliability of identification, we apply for this purpose the ICC’s *CW*1_*p*,*P*_(*t*_*n*_) (4) and *C*_*p*,*P*_(*t*_*n*_) (2) simultaneously. As an additional information, we use the correlation coefficients *c*_*s*,*S*_ (5) and integral correlation *C*_*s*,*S*_ (6) (see below) between the measured reflected THz signal *S*(*t*) and the standard THz signals *s*(*t*) transmitted through the most common explosives and illicit drugs under laboratory conditions.

A description of the physical experiment with PWM C4 is given in [29], [35], [37]. Reflected THz signals were registered on Teraview TPS 3000 setup in the standard reflection configuration [4] in a dry air with relative humidity less than 2%. The distance between the sample and detector is 30 cm. The weight of the pellet with PWM C4 is 600 mg. The Reference to calculate the stand-off reflectance was a gold mirror reflecting more than 99% of incident radiation [29].

Measurements of explosives RDX, HMX, PETN and TNT were made in transmission mode also using the Teraview TPS 3000 unit in the standard configuration. The distance between a sample and the receiver did not exceed 30 cm. Measurement procedure is described in detail in [4], but in our case, the RDX signal was measured in the ambient air.

The measurements of THz signals transmitted through the samples with illicit drugs MA, MDA, MDMA, Ketamine were made by using a Ti:Sapphire laser (Mai Tai Spectra-Physics Inc.). The details of the experiment can be found in [40].

### 3.1 Rough surface

**Fig 1:**
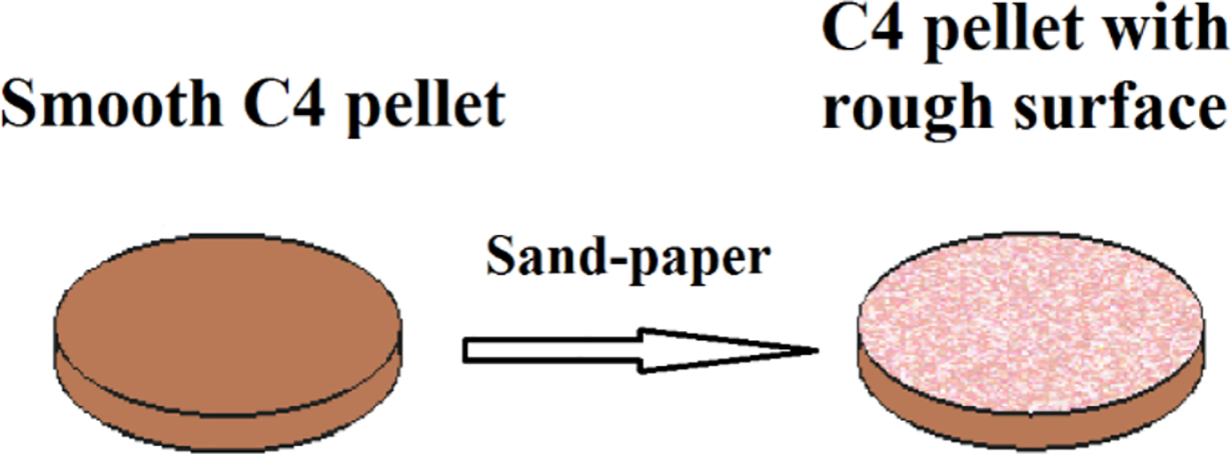
PWM_C4 pellet with rough surface.

In Fig 1 the PWM_C4 pellet with rough surface is shown. The surface was treated with sand paper grit 120, 80 and 40.

**Fig 2:**
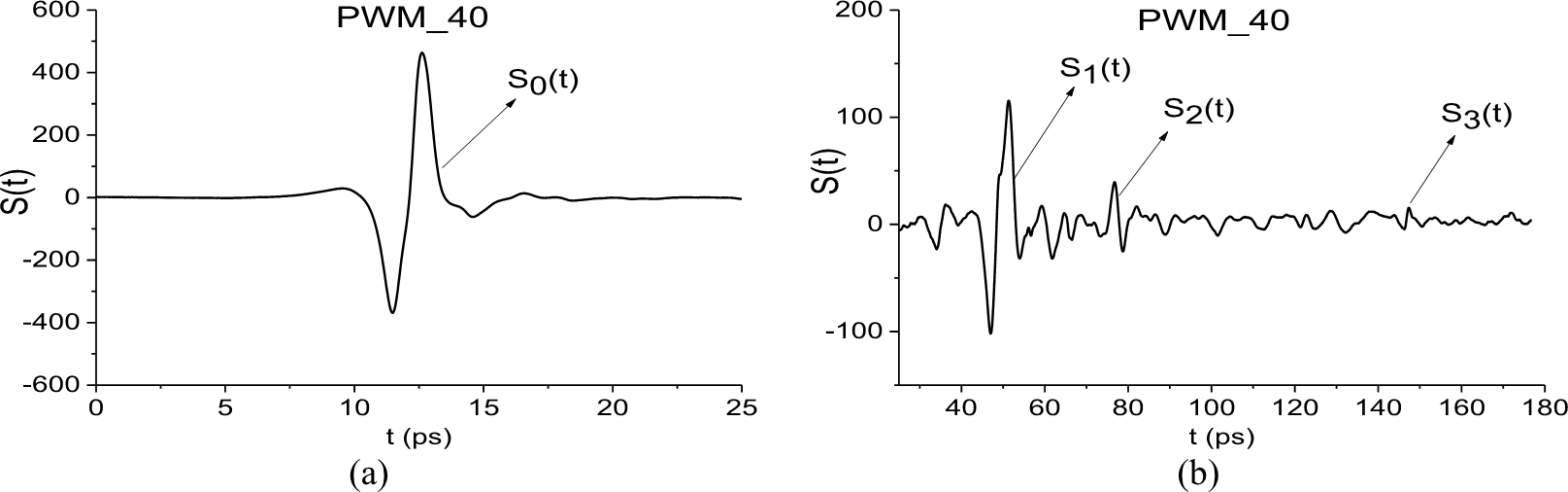
THz signal PWM_40 reflected from the rough surface in the time intervals (a) t = [0, 25] ps, (b) t = [25, 180] ps.

The THz signals reflected from the pellets with PWM C4, have duration about 180 ps. We denote them as PWM_120, PWM_80, PWM_40 signals for rough surfaces and the PWM signal for the initial smooth surface. In Fig 2 we present the PWM_40 signal in two time intervals: t = [0, 25] (a) and t = [25, 180] (b). The structure of the PWM_40 signal is clearly seen in this figure–it contains the pronounced main pulse *S*_0_(*t*) (a) reflected from the outer surface of the sample, the first sub-pulse *S*_1_(*t*) (b) reflected from the inner surface of the sample, and the consequent sub-pulses with significantly less amplitude *S*_2_(*t*), *S*_3_(*t*) (b) due to multiple reflection from inner surfaces. The rough PWM_120, PWM_80 and smooth PWM signals have the same structure.

#### 3.1.1. Spectral properties of the main pulses in the time interval t = [0, 25] ps

In this section, we present spectral properties of the main pulses of the reflected signals PWM and PWM_40, 80, 120, which partly were investigated in [29], [35], [37]. These results are necessary for further analysis of these THz signals by means of the ICC.

**Fig 3:**
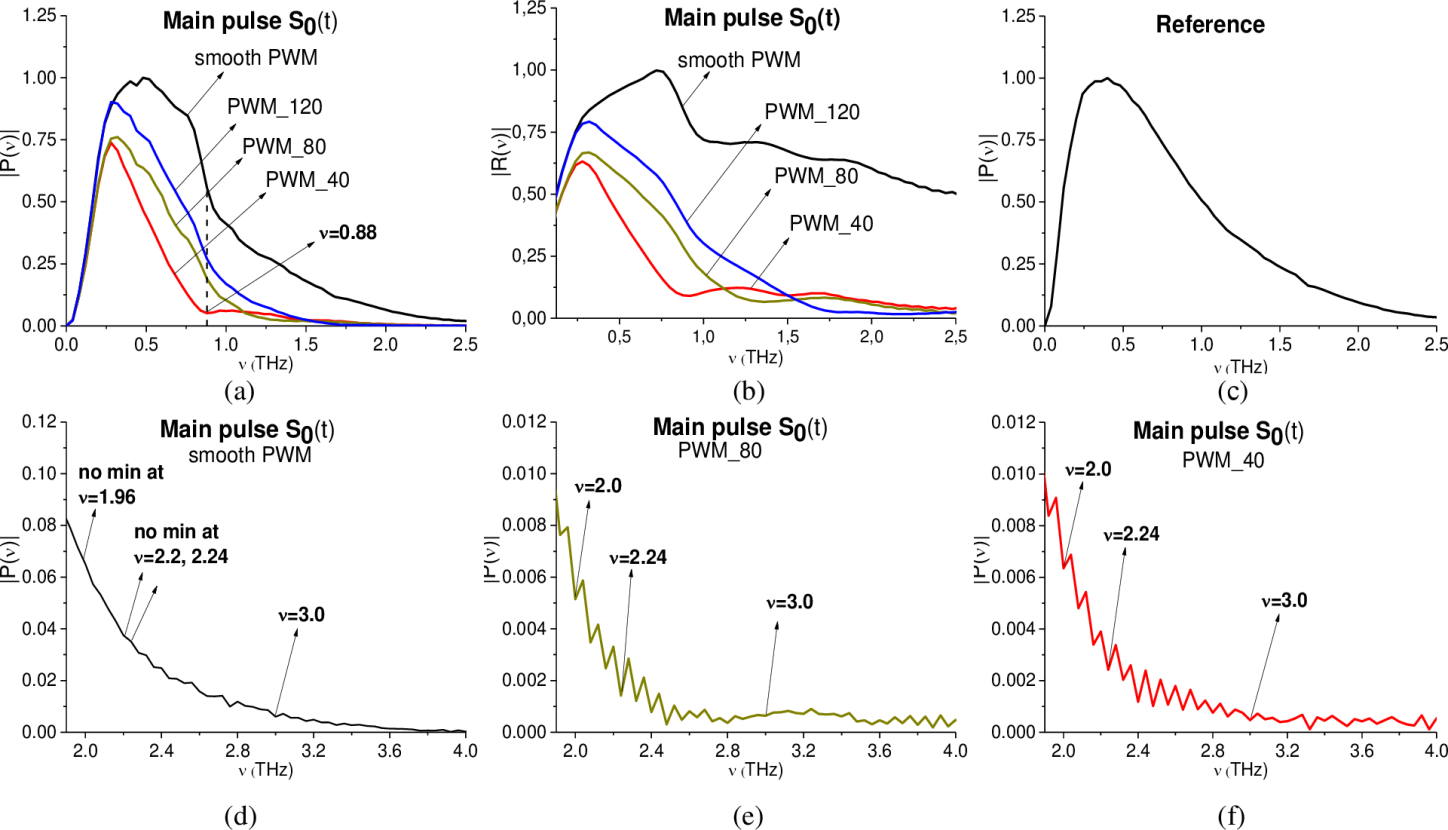
(a) Fourier spectra and (b) reflectance of the main pulses *S*_0_(*t*) of the PWM_40, PWM_80, PWM_120 and PWM reflected THz signals. (c) Reference signal spectrum. The spectra of the (d) smooth PWM, (e) PWM_80 and (f) PWM_40 in the frequency range *ν* = [2.0, 4.0] THz. The corresponding spectral resolution Δ*ν* = 0.04 THz.

As usual, the main pulse of the reflected THz signal is the object of the investigation and further analysis by means of the standard THz TDS. In Fig 3 the Fourier spectrum (a) and reflectance (b) of the main pulse *S*_0_(*t*) of smooth PWM and PWM_120, PWM_80, PWM_40 THz signals are depicted in the frequency range *ν* = [0.0, 2.5] THz. Reflectance *R*(*ν*) is calculated in the commonly used way as ratio *R*(*ν*) = |*P*(*ν*)|/|*P*_*REF*_(*ν*)|. Here |*P*(*ν*)|, |*P*_*REF*_(*ν*)| are the spectral amplitudes modulus of the main pulse of the measured PWM signal and Reference signal, correspondingly. In Fig 3(C) the Fourier spectrum of the reference signal calculated in the same time interval t = [0, 25] ps is depicted, it does not contain any pronounced spectral minima due to the environment influence. The frequency resolution for Fig 3 is equal to Δ*ν* = 0.04 THz. All figures (a)-(c) for convenience are shown in the same scale.

One can see that the shape of both Fourier spectra and reflectance of the PWM_40, 80, 120 signals differs greatly from spectra and reflectance of the smooth PWM signal. At the same time, in the reference spectrum (c), the spectral minima corresponding to the environment influence (including water vapor absorbance) are absent. Therefore, the spectral properties of the main pulse are distorted by the influence of the inhomogeneous surface only.

Recall that the substance RDX has the following absorption frequencies: ν = 0.82, 1.05, 1.36, 1.54, 1.95, 2.19, 3.0 THz [4], [5]. The pronounced RDX absorption frequencies are absent for the smooth PWM and PWM_120, 80, 40 signals in the frequency range *ν* = [0.0, 1.9] THz (a), (b). At the same time, in the PWM_40 spectrum (a) one can see the minimum at the frequency ν = 0.88 THz, which is close to RDX absorption frequency ν = 0.82 THz. In (d)-(f) the spectra of the smooth PWM, PWM_80, 40 signals are depicted in the frequency range *ν* = [1.9, 4.0] THz. One can see a single weak minima at the frequency ν = 3.0 THz for the smooth PWM spectrum (d), and three minima at the frequencies ν = 2.0, 2.24, 3.0 THz (close or equal to the RDX absorption frequencies ν = 1.95, 2.19, 3.0 THz) in the PWM_80, PWM_40 spectra (e), (f). The same minima are observed in the PWM_120 spectrum (not shown). In our opinion, this is because these THz signals contain a number of responses reflected from the rough surface at different angles, and the signals are averaged over these angles. Recall that each registered THz signal is a result of averaging over several realizations. In our case, the number of realizations is equal to 16 or 32. It is also worth to note that higher absorption frequencies are less distorted by inhomogeneity of the surface than lower frequencies.

However, this is not enough for the detection and identification of the plastic explosive PWM C4 by means of the standard THz TDS method. For this purpose, we will use the ICC’s. In the next sections, we will analyze the THz signal PWM_40 as the corresponding sample possesses the maximal roughness.

#### 3.1.2. Analysis of the correlation coefficients between the signal PWM_40 and the standard THz signals

In this section, we will examine how similar the signal PWM_40 and the standard signals from various hazardous substances are and how useful is this information for identification. For this purpose, we will assess the correlation coefficients between the reflected THz signal PWM_40 and the standard THz signals transmitted through the most common explosives and illicit drugs. In order to do this, we move the standard signal *s*(*t*) = {|*s*(*t*_*m*_)|}, *m* = 1,…,*N*_1_ along the long reflected signal *S*(*t*) = {|*S*(*t*_*m*_)|}, *m* = 1,…,*N*_2_. Here *N*_1_ and *N*_2_ are the numbers of time moments in these signals. Then, we calculate the time-dependent correlation coefficient *c*_*s*,*S*_(*t*_*n*_) (1), where instead of sets *p*_*ν*_ and *P*_*ν*_ we use the sets *s*(*t*_*m*_) and *S*(*t*_*m*+*n*_):

$$c_{s,S}(t_{n})=\frac{\sum_{m=0}^{N_{1}-1}\left(\left|s_{AV}(t_{m})|\cdot |S_{AV}(t_{m+n})\right|\right)}{\left\|s_{AV}(t_{m})\|\cdot \|S_{AV}(t_{m+n})\right\|},$$ (5)

where $s_{AV}(t_{m})=s(t_{m})-\bar{s},\;S_{AV}(t_{m+n})=S(t_{m+n})-\bar{S}$, $\bar{s}=\sum_{m=0}^{N_{1}-1}\left|s(t_{m})\right|/N_{1}$, $\bar{S}=\sum_{m=0}^{N_{1}-1}\left|S(t_{m+n})\right|/N_{1}$.

As the standard signals from explosives, we will use the transmitted THz signals RDX_Air, HMX_Air, PETN_Air and TNT_Air [31]. The standard signals from illicit drugs are the main pulses of the signals MA, MDA, MDMA and Ketamine [28], [33].

**Fig 4:**
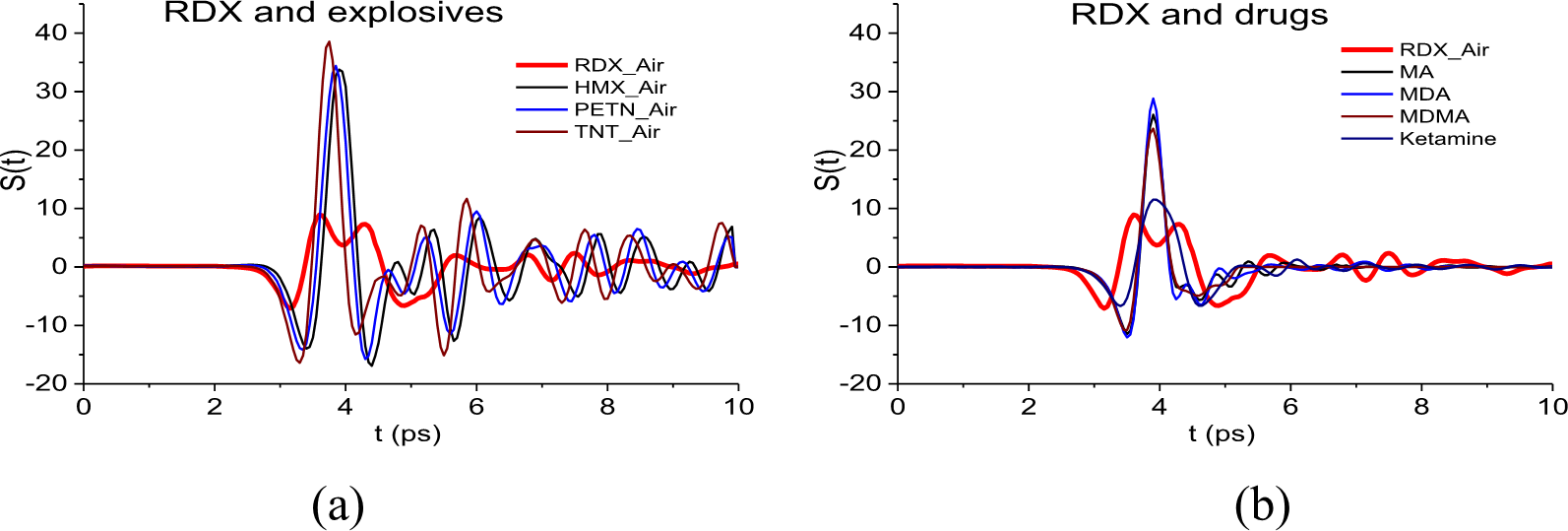
Standard THz signals (a) RDX_Air, HMX_Air, PETN_Air, TNT_Air; (b) MA, MDA, MDMA and Ketamine.

To avoid the influence of different signal length on correlation, all standard signals have the same duration 10 ps and similar structure–their maxima are located in the time interval t = [3, 5] ps. These are depicted in Fig 4(A) and 4(B). To start the calculations of the correlation coefficients *c*_*s*,*S*_(*t*_*n*_), we combine the corresponding signals beginnings at the point t = 0 ps.

**Fig 5:**
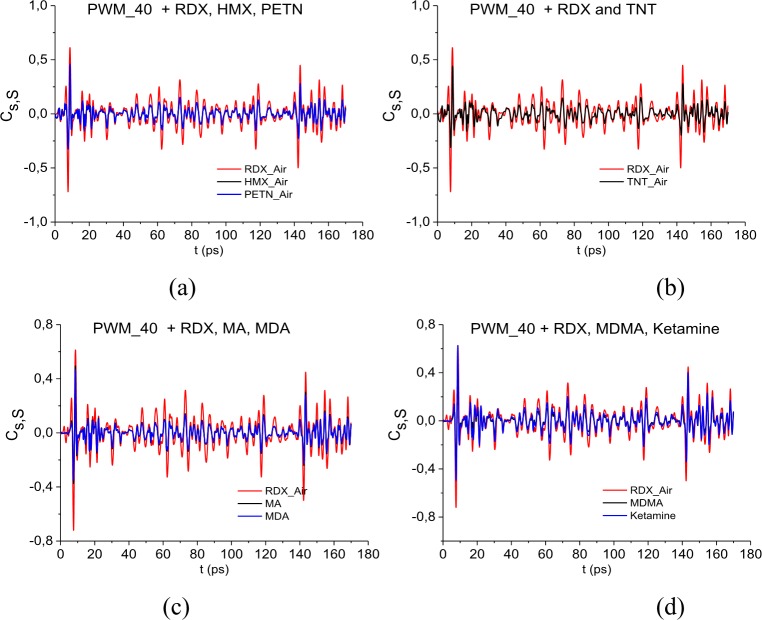
Correlation coefficients between the signal PWM-40 and the standard signals (a) RDX_Air, HMX_Air, PETN_Air, (b) RDX_Air, TNT_Air, (c) RDX_Air, MA, MDA, (d) RDX_Air, MDMA, Ketamine.

Fig 5 presents the values of correlation coefficients *c*_*s*,*S*_(*t*_*n*_) between the long reflected signal PWM_40 and the standard signals for explosives RDX_Air, HMX_Air, PETN_Air (a), as well as signal RDX_Air, TNT_Air (b). In Fig 5(C) and 5(D) the correlation coefficients *c*_*s*,*S*_(*t*_*n*_) (5) are obtained for the signal PWM_40 and the standard THz signals from illicit drugs MA, MDA (c), MDMA, Ketamine (d). The correlation coefficients for the signal RDX_Air are presented in (b), (c), (d) for comparison. One can see in (a)-(c) that the pronounced maxima of correlation coefficients occur in in the time intervals *t* = [0, 20] ps, [140, 170] ps, which correspond to the main pulse *S*_0_(*t*) and the last sub-pulse *S*_3_(*t*) of the signal PWM_40, see Fig 2.

In the time intervals t = [40, 60] ps and [60, 80] ps, which correspond to the sub-pulses *S*_1_(*t*), *S*_2_(*t*), the maximal values of the correlation coefficients *c*_*s*,*S*_(*t*_*n*_) obtained for the signals PWM_40 and RDX_Air are also greater than the corresponding maxima for PWM_40 and HMX_Air, PETM_Air (a), TNT_Air (b), MA, MDA (c) and MDMA, Ketamine (d).

The same results are observed for the PWM_80 and PWM_120 signals. In our opinion, it is because in the spectra of the PWM_40, 80, 120 main pulses there are at least three RDX absorption frequencies (and four—in the PWM_40 spectrum) while in the spectrum of the smooth PWM main pulse there is only one of them, ν = 3.0 THz.

By analogy with the integral correlation coefficient *C*_*p*,*P*_(*t*_*n*_) (3) for two spectral line dynamics, we can use the following integral characteristic for two signals:

$$C_{s,S}^{}(t_{n})=\sum_{m=0}^{n}|c_{s,S}(t_{m})|,\;n=0,\ldots ,M_{2}-M_{1}.$$ (6)

It allows us to better estimate the degree of similarity of the reflected THz signal PWM_40 and the standard THz signals from explosives and drugs.

**Fig 6:**
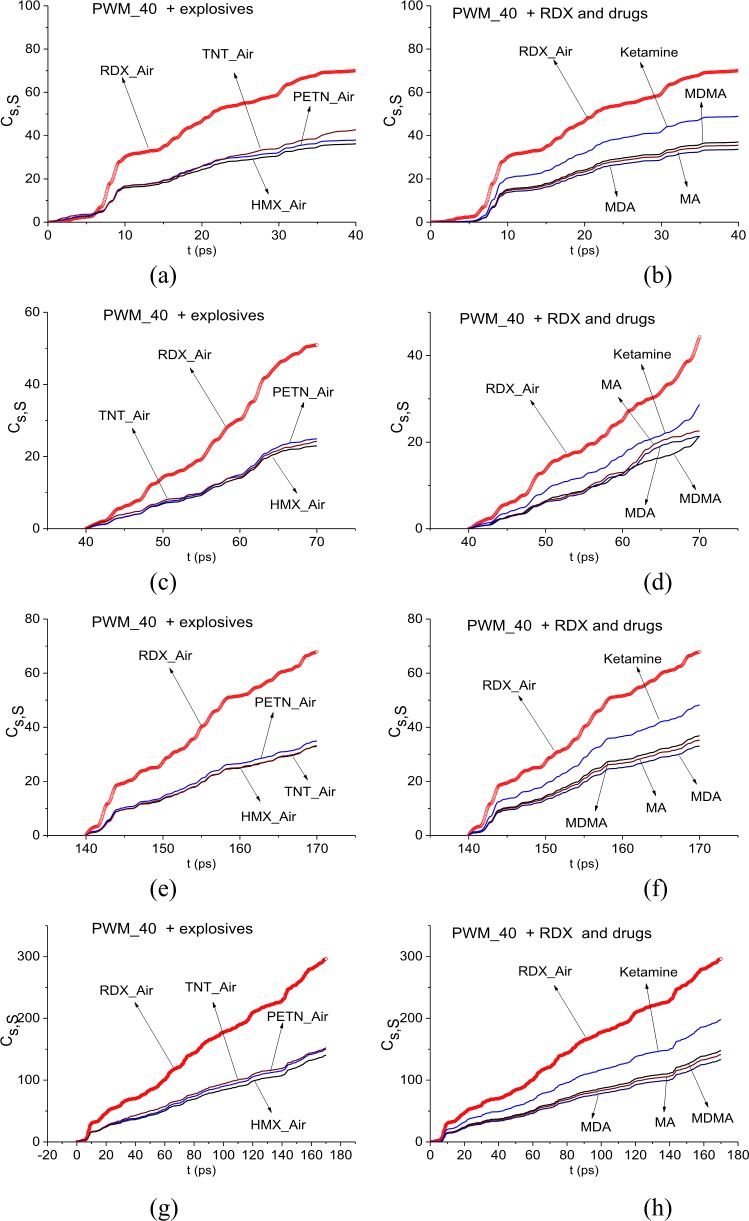
ICC *C*_*s*,*S*_ for the signal PWM_40 and the standard signals (a), (c), (e) RDX_Air, HMX_Air, PETN_Air, TNT_Air and (b), (d), (f) MA, MDA, MDMA, Ketamine signals in the time intervals (a), (b) t = [0, 40] ps, (c), (d) [40, 70] ps, (e), (f) [140, 170] ps and (g), (h) [0, 180] ps.

In Fig 6 the integral correlation criterion *C*_*s*,*S*_ is shown for the signal PWM_40 and the standard signals RDX_Air, HMX_Air, PETN_Air, TNT_Air (a), (c), (e) and MA, MDA, MDMA, Ketamine signals (b), (d), (f) in the time intervals t = [0, 40] ps (a), (b), [40, 70] ps, (c), (d), [140, 170] ps (e), (f). These time intervals contain the main pulse, the first and the last sub-pulses of the signal PWM_40, correspondingly. In (g), (h) the ICC *C*_*s*,*S*_ is depicted in the full time interval t = [0, 180] ps for the same signals. In all cases (a)-(h), the integral correlation between the signal PWM_40 and the standard signal RDX_Air is greater than for other standard signals from explosives and drugs. The same results are valid for the signals PWM_80 and PWM_120.

This fact indicates that degree of similarity between the signal PWM_40 and the standard signal RDX_Air is greater than the similarity between the PWM_40 signal and other standard signals from explosives and drugs not only for the first and the last sub-pulses, but over the entire time interval of measurement. However, it is not enough to detect the substance RDX, so in the next section we investigate the signal PWM_40 spectral properties and use the ICC’s.

#### 3.1.3. Analysis in the time interval t = [0, 25] ps, containing the main pulse

In [37], [38] we showed that the ICC *CW1*_*p*,*P*_ (4) allows us to be less dependent on noisy THz signal characteristics and to use for the substance detection and identification only the spectral characteristics of the standard signal. Below we show that this ICC can be applied to the main pulses of the reflected signals.

**Fig 7:**
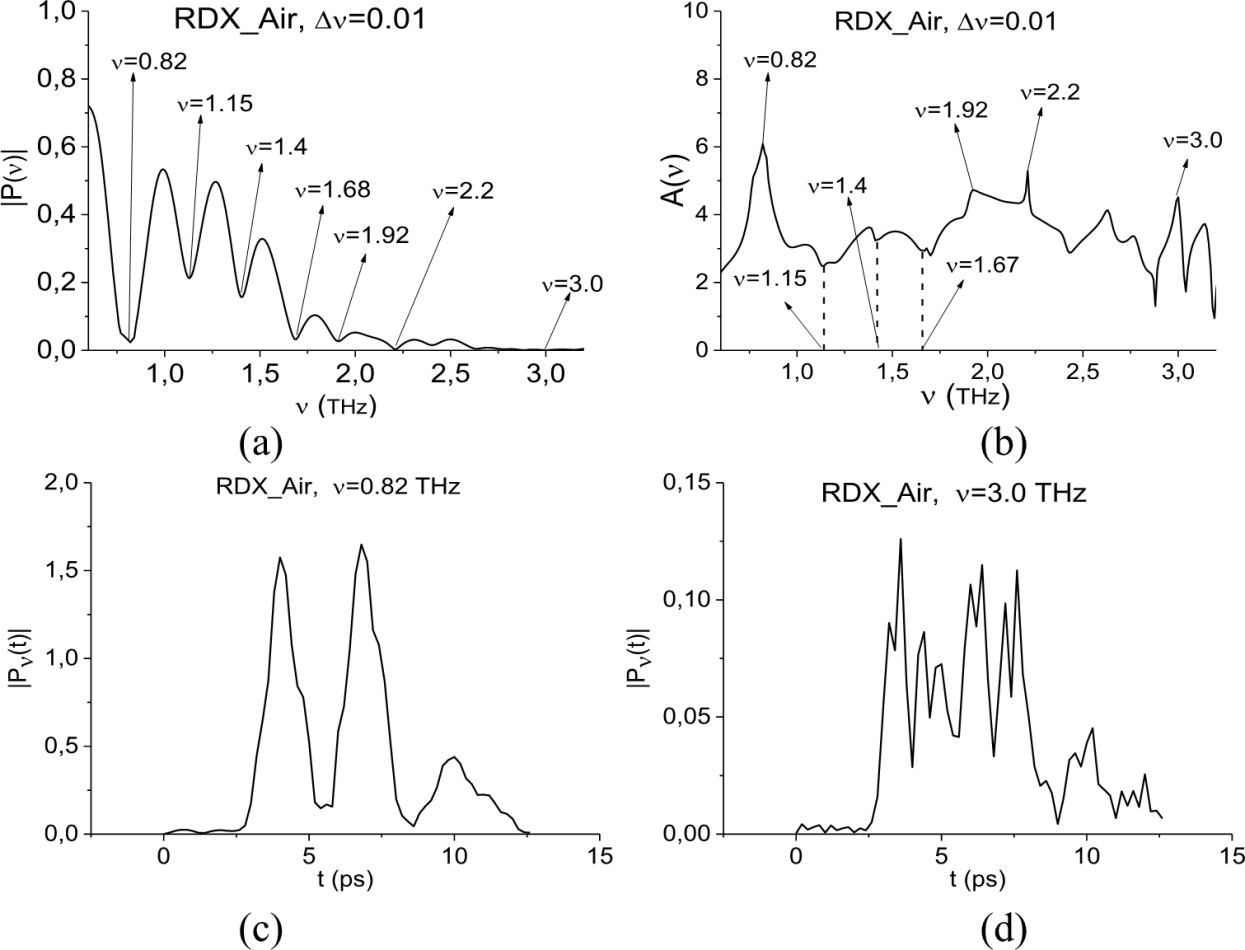
(a) Fourier spectrum and (b) absorbance of the standard THz signal RDX_Air. Time-dependent evolution of the spectral intensity at frequencies (c) ν = 0.82 THz; (d) 3.0 THz.

To find RDX absorption frequencies in the PWM_40 main pulse, we use the transmitted THz signal RDX_Air as the standard one and spectral lines dynamics of this signal at frequencies ν = 0.82, 3.0 THz. The frequency ν = 0.82 THz is the most known and bright RDX absorption frequency, ν = 3.0 THz is a brightest high absorption frequency. In Section 3.1.1 we showed the presence of minima at frequencies ν = 0.88 THz (close to ν = 0.82 THz) and 3.0 THz in the PWM_40 main pulse spectrum. The shape of the RDX_Air signal is shown in Fig 4; its Fourier spectrum, absorbance and corresponding dynamics of spectral lines can be found in Fig 7. The frequency resolution in (a), (b) is equal to Δν = 0.01 THz. In [35] we showed that the Fourier spectrum minima of the signal RDX_Air at frequencies ν = 1.15, 1.4 THz do not correspond to absorption frequencies of pure RDX. They are caused by water vapor containing in the air during the measurement and cannot be used for the detection and identification.

**Fig 8:**
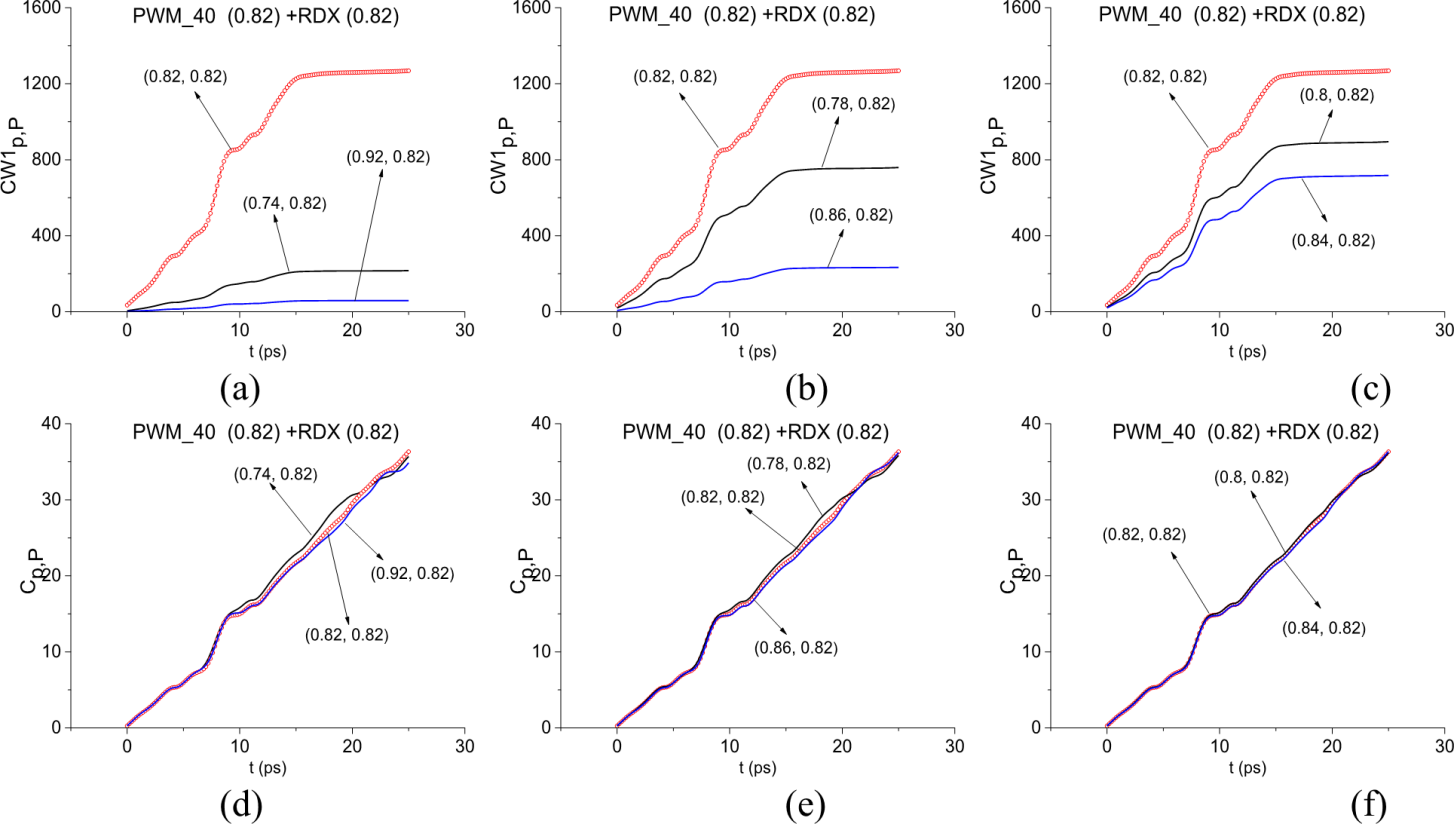
(a)-(c) Time-dependent ICC *CW*1_*p*,*P*_, (d)-(f) *C*_*p*,*P*_ calculated for the frequency ν = 0.82 THz in the frequency ranges (a), (d) *ν* = [0.74, 0.92] THz, (b), (e) [0.78, 0.86] THz, (c), (f) [0.8, 0.84] THz.

In Fig 8 the ICC *CW1*_*p*,*P*_ (a)-(c) and *C*_*p*,*P*_ (d)-(f) are calculated for the frequency *ν* = 0.82 THz in the reducing frequency ranges *ν* = [0.74, 0.92] THz (a), (d), [0.78, 0.86] THz (b), (e), [0.8, 0.84] THz (c), (f). In all cases (a)-(c) the frequency *ν* = 0.82 THz is detected by the ICC *CW1*_*p*,*P*_. By decreasing the detection range, the contrast of detection is also decreases. At the same time, the ICC *C*_*p*,*P*_ does not demonstrate detection in these frequency ranges in the whole time interval t = [0, 25] ps, but detection is observed partly in the time interval t = [0, 9] ps in (d), in t = [0, 10] ps in (e) and t = [0,15] ps in (f). That is, we found a frequency range, *ν* = [0.8, 0.84] THz, in which both criteria simultaneously detect the frequency *ν* = 0.82 THz as the RDX absorption frequency in the PWM_40 main pulse. We will call this frequency range as a minimal FDR.

**Fig 9:**
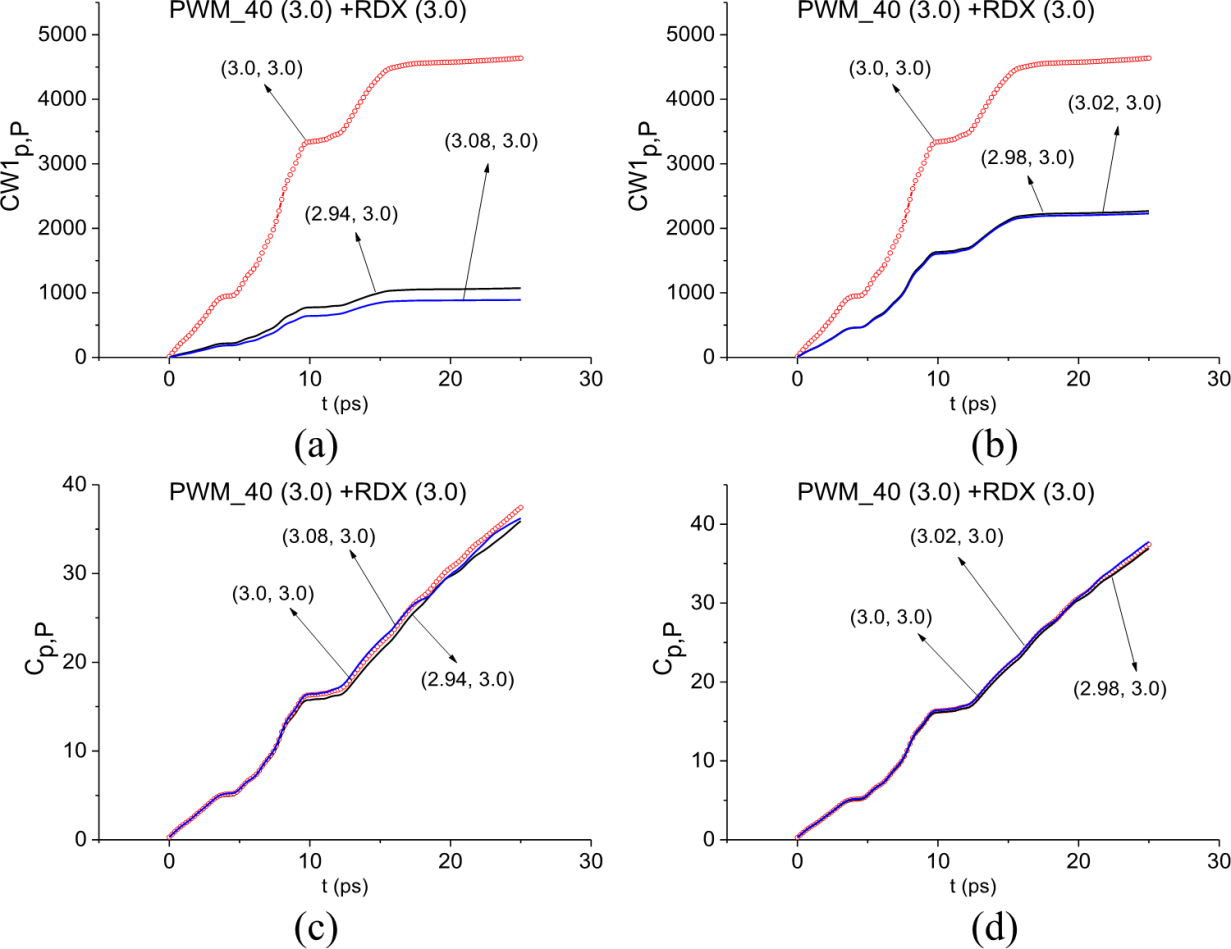
(a), (b, Time-dependent ICC *CW*1_*p*,*P*_, (c), (d) *C*_*p*,*P*_ calculated for the frequency ν = 3.0 THz in the frequency ranges (a), (b) *ν* = [2.94, 3.08] THz, (c), (d) [2.8, 3.02] THz.

In Fig 9 the ICC *CW1*_*p*,*P*_ (a), (b) and *C*_*p*,*P*_ (c), (d) are calculated for the frequency *ν* = 3.0 THz in the reducing frequency ranges *ν* = [2.94, 3.08] THz (a), (b), [2.98, 3.02] THz (c), (d). In the cases (a), (b) the frequency *ν* = 3.0 THz is detected by the ICC *CW1*_*p*,*P*_. As in the case of the frequency *ν* = 0.82 THz detection, the contrast of detection decreases with decreasing the detection range. Detection with the ICC *C*_*p*,*P*_ is observed partly in the time interval t = [0, 10] ps in (c), in t = [0, 15] ps in (d). So, the minimal FDR for the frequency *ν* = 3.0 THz is *ν* = [2.98, 3.02] THz.

Therefore, the ICC *CW*1_*p*,*P*_ and *C*_*p*,*P*_ detect the frequencies *ν* = 0.82, 3.0 THz as RDX absorption frequencies in the main pulse of the signal PWM_40. Taking also into account the results obtained in Section 3.1.1 with the help of the ICC *C*_*s*,*S*_, we can conclude about the presence of explosive RDX absorption frequencies in the main pulse of the signal PWM_40.

#### 3.1.4. Analysis in the time interval t = [40, 65] ps, containing the first sub-pulse

**Fig 10:**
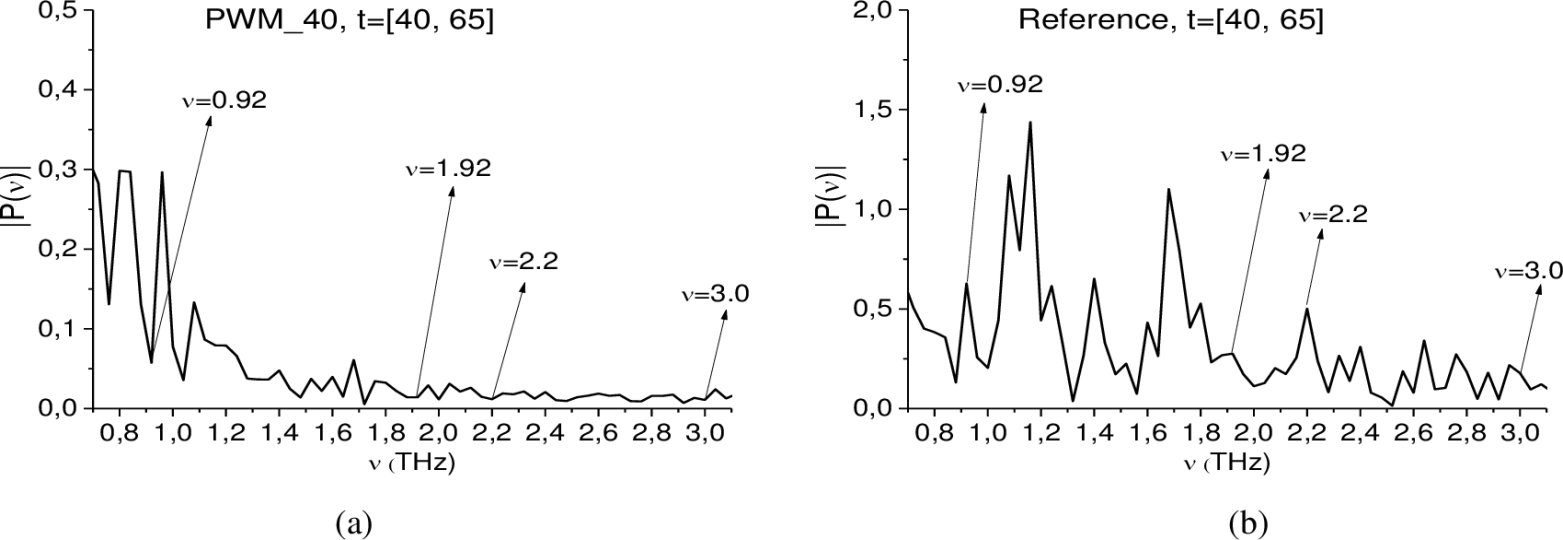
Fourier spectra of the (a) PWM_40 first sub-pulse and (b) reference signal spectrum in the frequency range ν = [0.7, 3.1] THz.

In this section, we show the possibility of PWM C4 identification using a time interval, containing the first sub-pulse *S*_*1*_*(t)* of the reflected THz signal, by means of the ICC’s *CW*1_*p*,*P*_(*t*_*n*_) and *C*_*p*,*P*_(*t*_*n*_). Fig 10 shows the Fourier spectrum of the signal PWM_40 (a) and the reference signal (b) in the frequency range ν = [0.7, 3.1] THz. They are calculated in the time interval t = [40, 65] ps with frequency resolution ν = 0.04 THz.

The PWM_40 spectrum (b) contains minima (and Reference spectrum (c)–maxima) at the frequencies ν = 0.92, 1.92, 2.2. 3.0 THz, that are close to the absorption frequencies of explosive RDX ν = 0.82, 1.95, 2.19, 3.0 THz [4], [5]. The difference between these frequencies may be caused by the difference in frequency resolution (in our analysis Δν = 0.04 THz, in [4], [5] Δν = 0.01 THz) and influence of the inhomogeneous surface. Recall that in [35] these frequencies were detected as RDX absorption frequencies in the signal PWM_40 be means of the ICC *CW*_*p*,*P*_(*t*_*n*_). It should be noted that the minima corresponding to these frequencies, are absent in the reference signal spectrum (c) that is, absorption at frequencies in (b) are not caused by water vapor or environment influence. The minimum at frequencies, which are close to the RDX absorption frequency ν = 0.82 THz, is also absent in (b).

**Fig 11:**
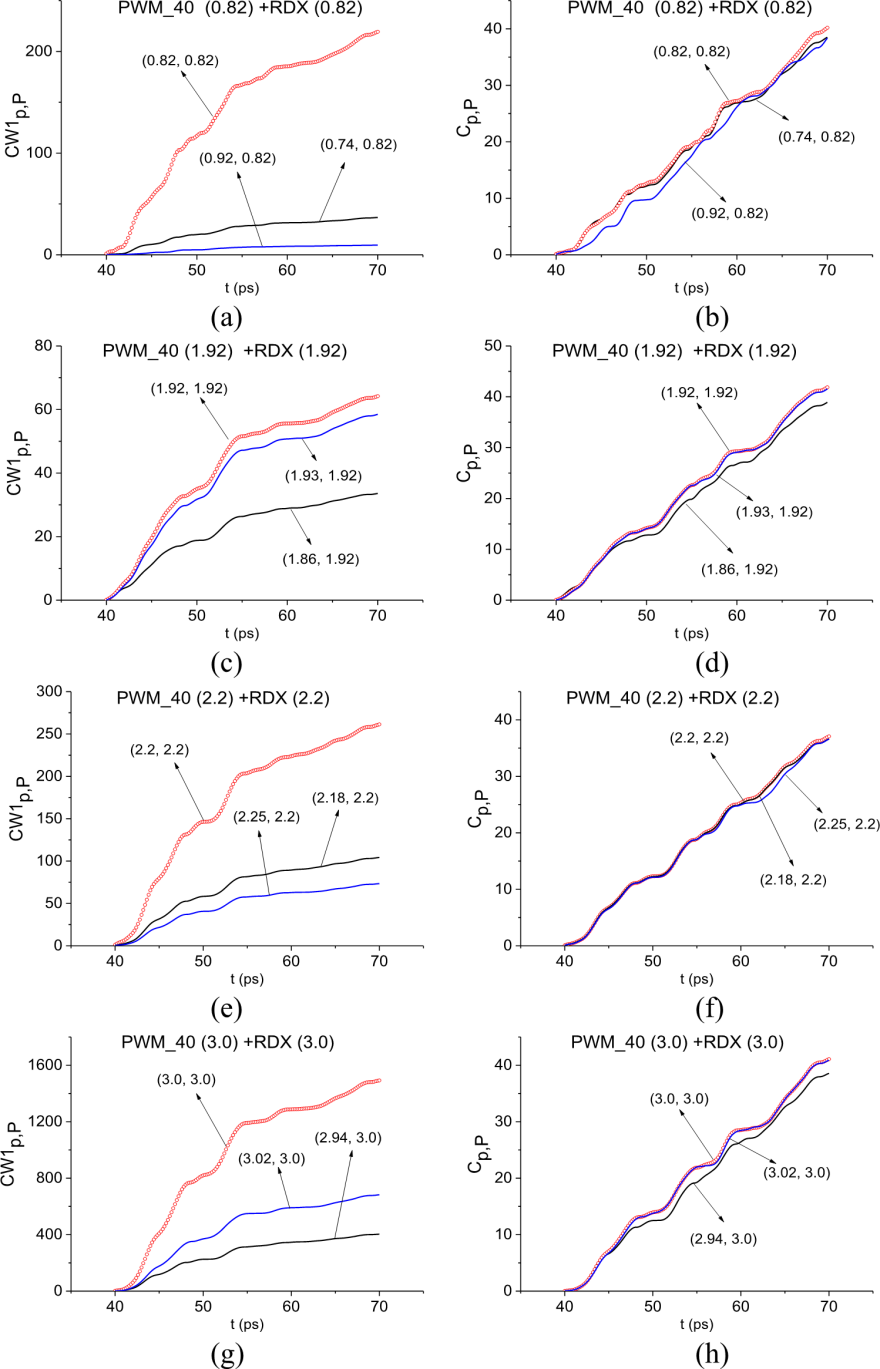
ICC *CW*1_*p*,*P*_(*t*_*n*_) and *C*_*p*,*P*_(*t*_*n*_) detecting frequencies (a), (b) ν = 0.82 THz 1.92 THz, (e), (f) 2.2 THz and (g), (h) 3.0 THz in the time interval t = [40, 70] ps.

In Fig 11 the ICC *CW*1_*p*,*P*_ and *C*_*p*,*P*_ evolution is depicted at frequencies ν = 0.82 THz (a), (b), 1.92 THz (c), (d), 2.2 THz (e), (f) and 3.0 THz (g), (h). The corresponding FRD’s are *ν* = [0.74, 0.92] THz (a) (b), [1.86, 1.93] THz (c), (d), [2.18, 2.25] THz (e), (f), and [2.94., 3.02] THz (g), (h).

In all cases, both ICC’s detect these frequencies as the absorption frequencies of RDX. Summarizing results obtained in Sections 3.1.4 and 3.1.5 for the signal PWM_40, we conclude that if the standard substance (in our case, this is explosive RDX) is present in the sample with a rough surface, then there exists a minimal FDR where both ICC’s *CW*1_*p*,*P*_ and *C*_*p*,*P*_ detect the absorption frequency of this substance in the investigated signal simultaneously.

#### 3.1.5. Absence of absorption frequencies of explosives HMX, PETN, TNT and illicit drug MA in PWM_40 signal

**Fig 12:**
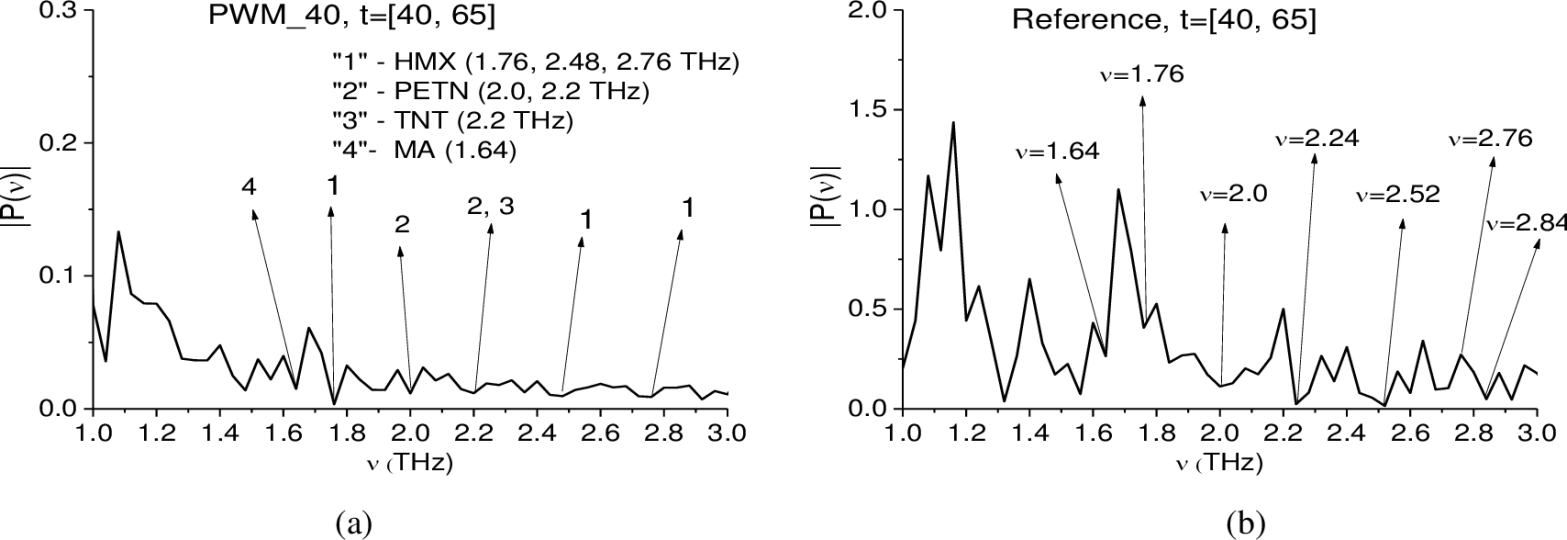
(a) The Fourier spectrum of the PWM_40 first sub-pulse and (b) the Reference signal in the time interval t = [40, 65] ps in the frequency range ν = [0.7, 3.1] THz.

Below we analyze the Fourier spectra of the PWM_40 and Reference signals simultaneously in the time interval t = [40, 65] ps as well as use ICC *C*_*p*,*P*_ in order to demonstrate the absence of absorption frequencies of other dangerous substances in the signal PWM_40. The Fourier (a) and Reference (b) spectra are depicted in Fig 12 in the frequency range *ν* = [1.0, 3.0] THz.

In Fig 12(A) one can see not only the minima corresponding the RDX absorption frequencies, but minima, which are close to the absorption frequencies of other explosives and drugs. Thus, minima at frequencies *ν* = 1.76, 2.48, 2.82 THz are close to HMX absorption frequencies, minima at *ν* = 2.0, 2.2 THz–to those of PETN, *ν* = 2.2 –to that of TNT [4], [5], *ν* = 1.64 THz is close to MA absorption frequency [37], [40]. At the same time, in the Reference spectrum (b) one can see minima at close frequencies ν = 1.64, 1.76, 2.0, 2.24, 2.52, 2.82 THz, which can be caused by the environment influence.

Therefore, simultaneous analysis of the PWM_40 and Reference signals spectra in the time interval t = [40, 65] ps allows us to exclude from consideration the frequencies *ν* = 1.76, 2.48, 2.82 THz (HMX), *ν* = 2.0, 2.2 THz (PETN), TNT, *ν* = 1.64 THz (MA), they are not the absorption frequencies of substances MA and PETN. Taking into account the fact that the remaining absorption frequencies of these substances are also absent in the spectrum (a), we conclude that these substances are absent in the sample with PWM_C4 explosive.

**Fig 13:**
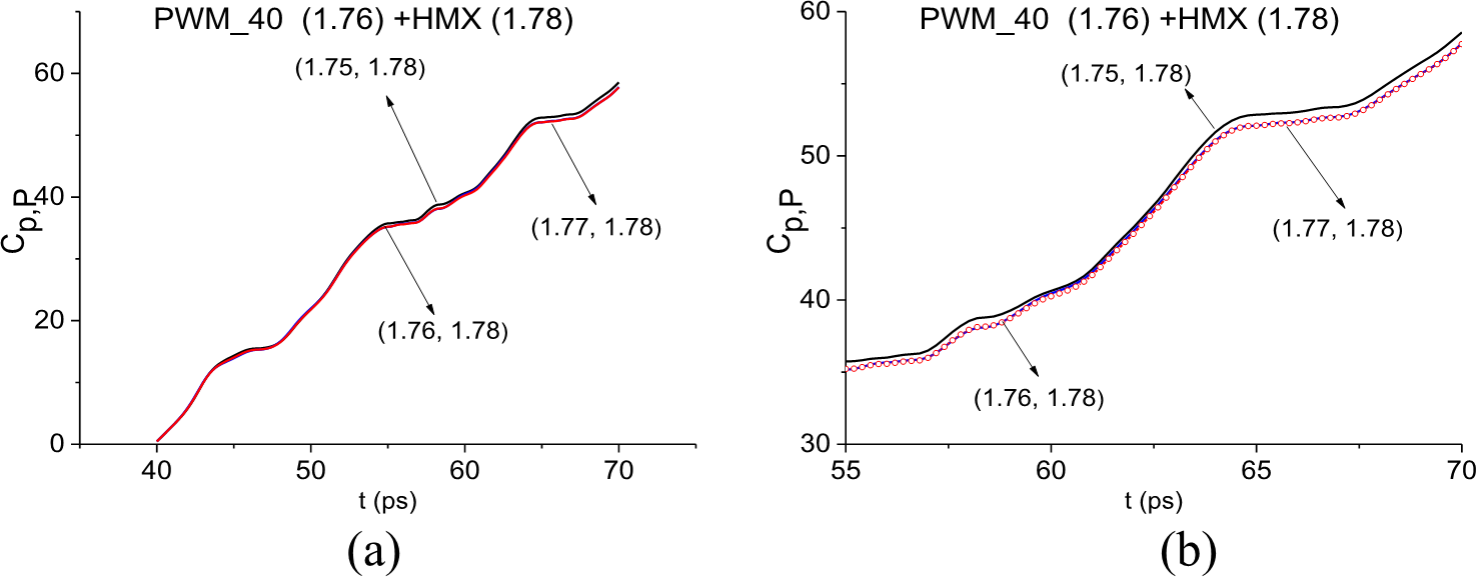
ICC *C*_*p*,*P*_(*t*_*n*_)calculated for the frequency ν = 1.76 THz with HMX_Air standard signal in the time intervals (a) t = [40, 70] ps,(b) t = [55, 70] ps.

The absence of HMX absorption frequency in the first sub-pulse of PWM_C4 signal, for example, *ν* = 1.76 THz, close to HMX absorption frequency *ν* = 1.78 THz can be also confirmed by using the ICC *C*_*p*,*P*_. The spectral line dynamics of the standard signal HMX_Air (see Fig 4) at the frequency *ν* = 1.78 THz was used as a standard one. In Fig 13 this ICC is shown in the FDR *ν* = [1.75, 1.77] THz in the time interval t = [40, 70] ps (a). The ICC *C*_*p*,*P*_ does not detect the frequency *ν* = 1.76 THz as HMX absorption frequency in the PWM_40 signal. In the shortened time interval t = [55, 70] ps (b) the line corresponding to the frequency *ν* = 1.76 THz is not the topmost.

The absence of TNT and MDMA absorption frequency in the PWM_C4 first sub-pulse can be shown in the same way. Thus, the use of the ICC *C*_*p*,*P*_(*t*_*n*_) together with analysis of the Reference spectrum can reduce the false positive and negative alarms.

#### 3.1.6. Analysis in the time interval t = [70, 170] ps

**Fig 14:**
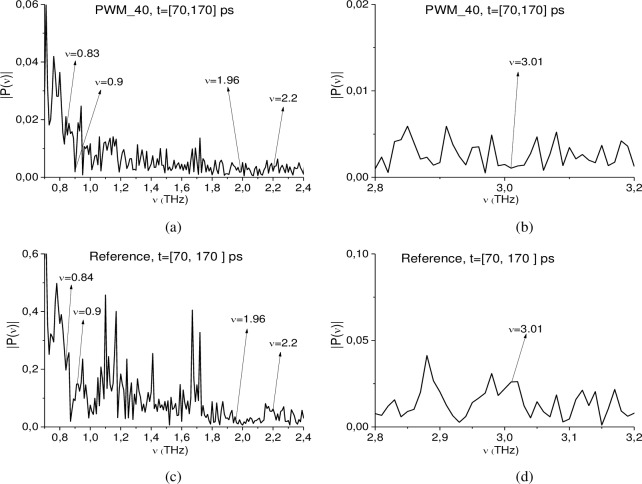
(a), (b) Fourier spectra of the PWM_40 signal and (c), (d) Reference signal calculated in the time interval t = [70, 170] ps in the frequency ranges (a), (c) ν = [0.7, 2.4] THz, (b), (d) [2.8, 3.2] THz.

In this section we show that one can identify plastic explosives with rough surface in the remote time interval *t* = [70, 170] ps, which contains two sub-pulses *S*_2_(*t*) and *S*_3_(*t*), using the ICC’s *CW*1_*p*,*P*_(*t*_*n*_) and *C*_*p*,*P*_(*t*_*n*_). Fig 14 shows the Fourier spectra of the PWM_40 (a), (b) and reference (c), (d) signals calculated in the time interval t = [70, 170] ps, for the frequency ranges ν = [0.7, 2.4] THz (b), (d), [2.8, 3.2] THz (d), (e). In Fig 14(B) and 14(C) one can see minima at frequencies ν = 0.9, 1.96, 2.2, 3.01 THz. In Fig 14(D) and 14(E) minima, corresponding to the absorption frequencies of the environment, are absent. As above, we use the spectral lines dynamics of the RDX_Air signal at frequencies ν = 0.82, 1.95, 2.2, 3.0 THz as the standard ones.

**Fig 15:**
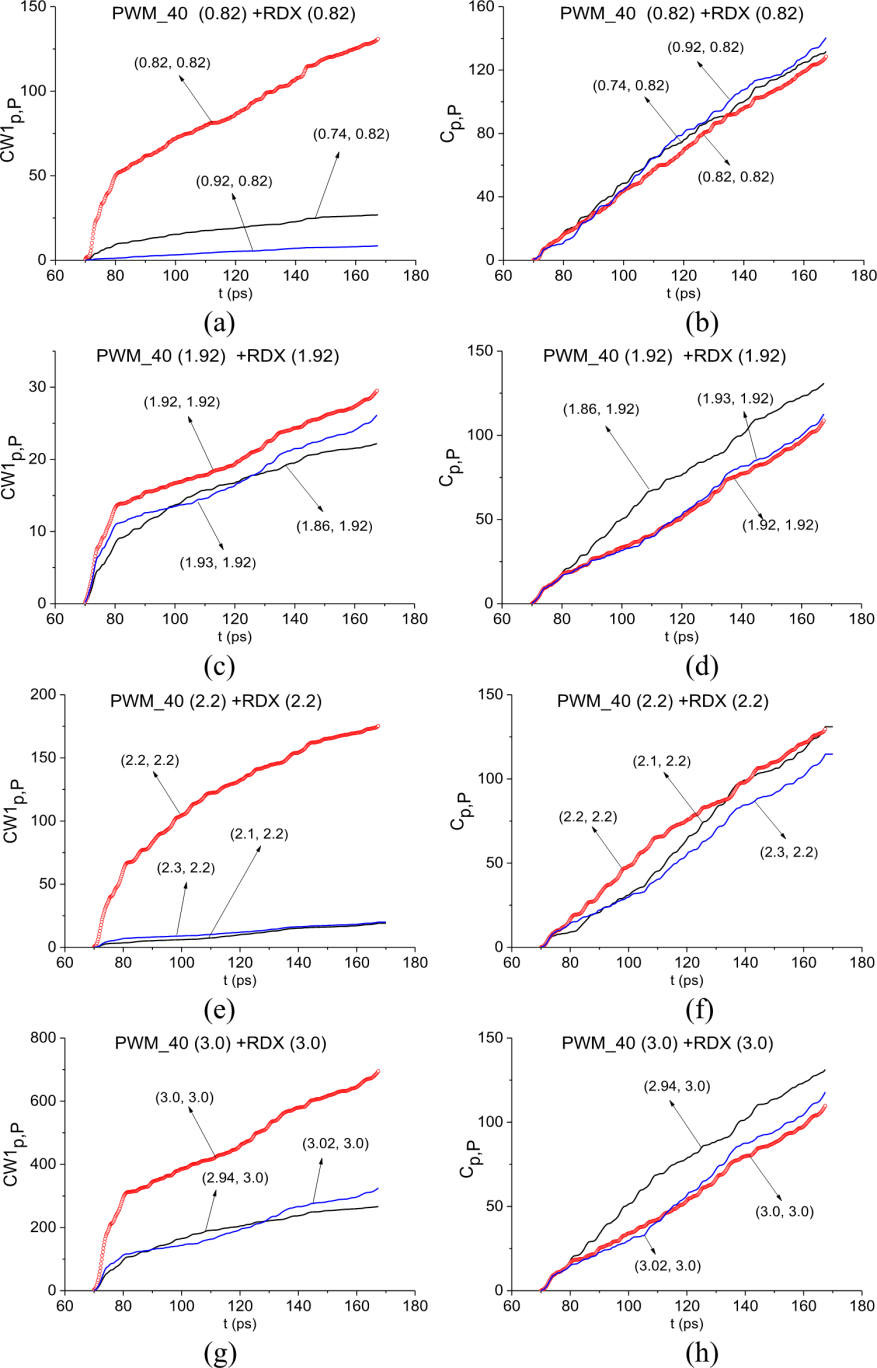
ICC *CW*1_*p*,*P*_(*t*_*n*_)and *C*_*p*,*P*_(*t*_*n*_) detecting frequencies (a), (b) ν = 0.82 THz, (c), (d) 1.92 THz, (e), (f) 2.2 THz and (g), (h) 3.0 THz in the time interval t = [70, 170] ps.

In Fig 15 the ICC *CW*1_*p*,*P*_ and *C*_*p*,*P*_ evolution is depicted at frequencies ν = 0.82 THz (a), (b), 1.92 THz (c), (d), 2.2 THz (e), (f) and 3.0 THz (g), (h). The corresponding minimal FDR is *ν* = [0.74, 0.92] THz (a) (b), [1.86, 1.93] THz (c), (d), [2.1, 2.3] THz (e), (f), and [2.94., 3.02] THz (g), (h). In all cases, the ICC *CW*1_*p*,*P*_ detects these frequencies as the absorption frequencies of RDX in the full time interval t = [70, 170] ps. At the same time, the ICC *C*_*p*,*P*_ detects frequencies ν = 0.82 THz (b), 1.92 THz (d) and 3.0 THz (h) only in the shortened time intervals corresponding to the sub-pulse *S*_2_(*t*). The frequency ν = 2.2THz is detected by the ICC *C*_*p*,*P*_ in the full time interval under investigation t = [70, 170] ps.

Analysis of the spectral properties of the last sub-pulse *S*_3_(*t*) in the short time interval t = [145, 168] ps length t = 23 ps shows that the ICC *CW*1_*p*,*P*_ also detects the frequencies *ν* = 0.82 THz (a), *ν* = 1.92 THz (b), *ν* = 2.2 THz (c), *ν* = 3.0 THz (d) as RDX absorption frequencies in this time interval (not shown). The corresponding minimal FRD’s are *ν* = [0.78, 0.92] THz (a), *ν* = [1.9, 1.93] THz (b), *ν* = [2.1, 2.3] THz (c), *ν* = [2.98, 3.02] THz (d).The maximal contrast is observed for the frequencies *ν* = 0.82 THz (a) and *ν* = 2.2 THz (c).

Therefore, the ICC’s *CW*1_*p*,*P*_(*t*_*n*_) (4) and *C*_*p*,*P*_(*t*_*n*_) (2) allow us to detect the RDX absorption frequencies in the PWM_40 signal both in the short time interval, containing the first sub-pulse, and in the remote time interval, which contains the following sub-pulses with less amplitude. The results obtained by using the ICC *C*_*s*,*S*_ (6), which assesses the similarity of the signal PWM_40 and the standard signal RDX_Air, are an additional confirmation of the RDX presence in the PWM_40 sample with rough surface. Note that for the reliable and effective substance detection is necessary to use several criteria simultaneously, including the ICC *C*_*p*,*P*_(*t*_*n*_). The only one criterion using may be insufficient for this purpose. The RDX absorption frequencies were detected in the PWM_80 and PWM_120 signals in the same way.

### 3.2 Concave surface

**Fig 16:**
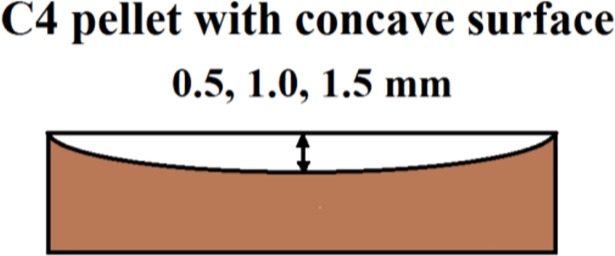
PWM C4 pellet with concave surface.

In this section, we continue our investigations of the spectral properties of the THz signals reflected from the PWM_C4 pellet with concave surface, which were given in [29], [35]. The curvature radius of the surface is 0.5, 1.0 or 1.5 mm, see Fig 16. We denote these THz signals as PWM_0.5, PWM_1.0 and PWM_1.5 signals. The signal structure remains the same as it was in Section 3.1, see Fig 2.

**Fig 17:**
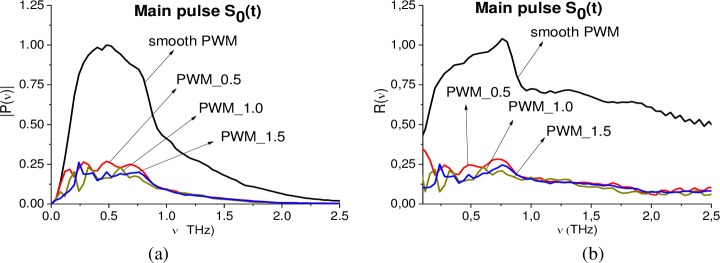
(a) Fourier spectra of the main pulses of the concave PWM_0.5, PWM_1.0, PWM_1.5 signals and smooth PWM signal in the frequency range *ν* = [0.0, 2.5] THz. (b) PWM_1.5 main pulse spectrum in the frequency range *ν* = [2.0, 4.0] THz.

Fig 17 shows the Fourier spectra (a) of the main pulse of the PWM_0.5, PWM_1.0, PWM_1.5 signals in the frequency range ν = [0.0, 2.5] THz. For comparison, the corresponding spectral characteristics for the smooth PWM signal are also given in (a). One can see the pronounced difference between the spectral amplitudes of the concave PWM_0.5, 1.0, 1.5 signals and smooth PWM signal in (a)–they are several times less than that of the smooth one. As well the shape of the corresponding spectra for concave and smooth PWM signals in Fig 17(A) significantly differs from each other. The pronounced absorption frequencies of the explosive RDX ν = 0.82, 1.05, 1.36, 1.54, 1.95, 2.19 THz (or minima close to them) [4], [5] are absent in (a) for all THz signals reflected from concave surface. In (b) the PWM_1.5, PWM_1.0, PWM_0.5 main pulse spectra are shown in the frequency range ν = [2.0, 4.0] THz. In the PWM_0.5 spectrum, minima at RDX absorption frequencies (or close to them) are absent. In PWM_1.0 and PWM_1.5 main pulse spectra there is a single minimum at the frequency ν = 3.04 THz, which is close the RDX absorption frequency ν = 3.0 THz. Consequently, concave surface also greatly distorts the spectral properties of the main pulses of the reflected THz signals, so that the standard THz TDS using is inefficient for the detection and identification. The corresponding spectral resolution is equal to ν = 0.04 THz.

#### 3.2.1. Analysis of the correlation coefficients between the signal PWM_1.5 and the standard THz signals

In this section, we assess the correlation coefficients *c*_*s*,*S*_(*t*_*n*_) (5) between the reflected THz signal PWM_1.5 and the standard THz signals transmitted through the explosives and illicit drugs. As in Section 3.1.1, we will use the transmitted THz signals RDX_Air, HMX_Air, PETN_Air and TNT_Air as well as signals MA, MDA, MDMA and Ketamine as the standard ones.

**Fig 18:**
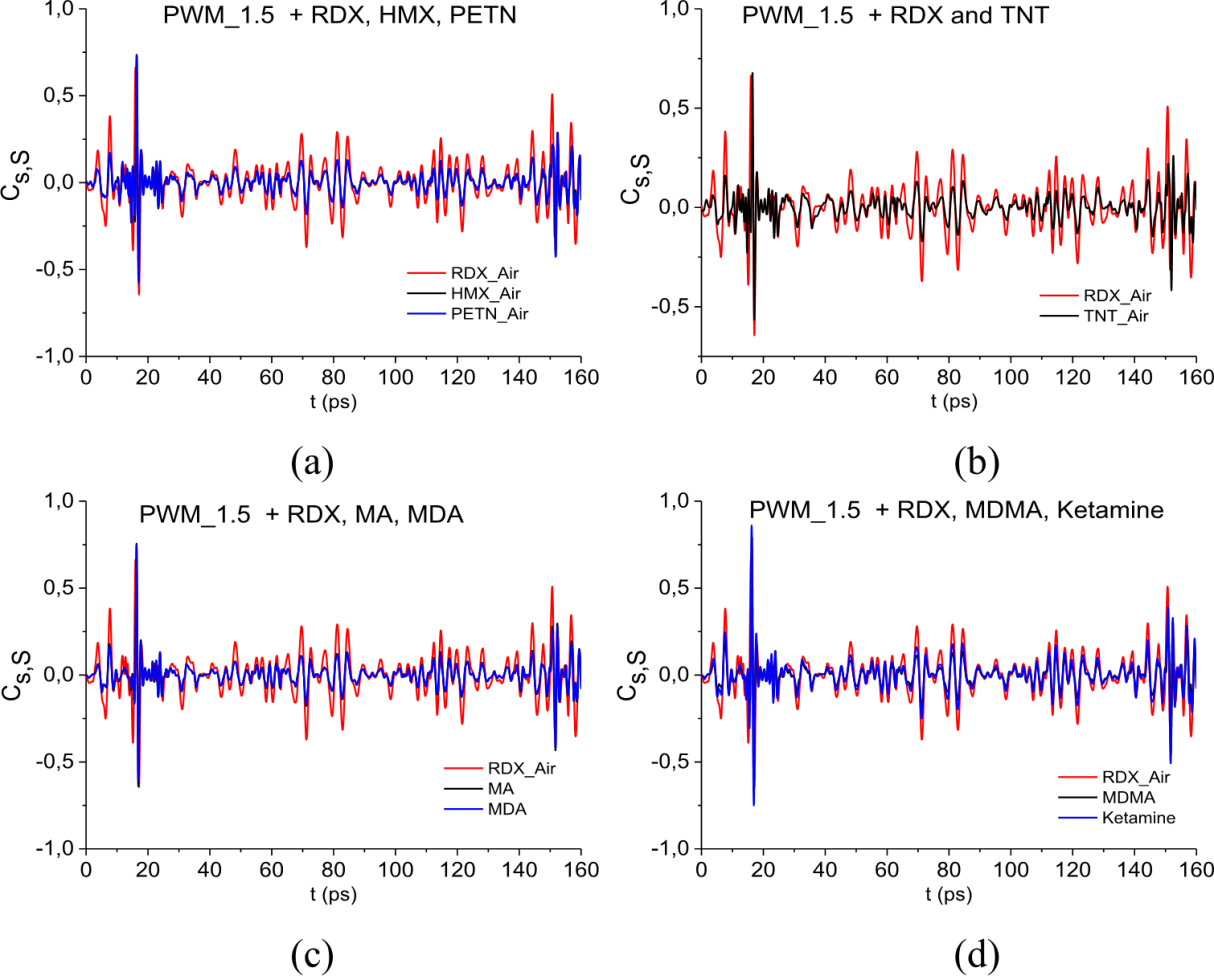
Correlation coefficients between the signal PWM-40 and the standard signals (a) RDX_Air, HMX_Air, PETN_Air,(b) RDX_Air, TNT_Air, (c) RDX_Air, MA, MDA, (d) RDX_Air, MDMA, Ketamine.

Fig 18 shows the correlation coefficients *c*_*s*,*S*_(*t*_*n*_) between the long reflected signal PWM_1.5 and the standard signals for explosives RDX_Air, HMX_Air, PETN_Air in (a) and RDX_Air, TNT_Air in (b). In Fig 18(C) and 18(D) the correlation coefficients *c*_*s*,*S*_(*t*_*n*_) (5) are obtained for the signal PWM_1.5 and the standard THz signals from illicit drugs MA, MDA (c), MDMA, Ketamine (d). The correlation coefficients for the signal RDX_Air are presented in (b)-(d) for comparison. The maximal values of the correlation coefficients *c*_*s*,*S*_(*t*_*n*_) obtained for the signals PWM_1.5 and RDX_Air are greater than the corresponding maxima for PWM_1.5 and HMX_Air, PETM_Air (a), TNT_Air (b), MA, MDA (c) and MDMA, Ketamine (d), in the time interval t = [25, 160] ps, which does not contain the PWM_1.5 main pulse.

**Fig 19:**
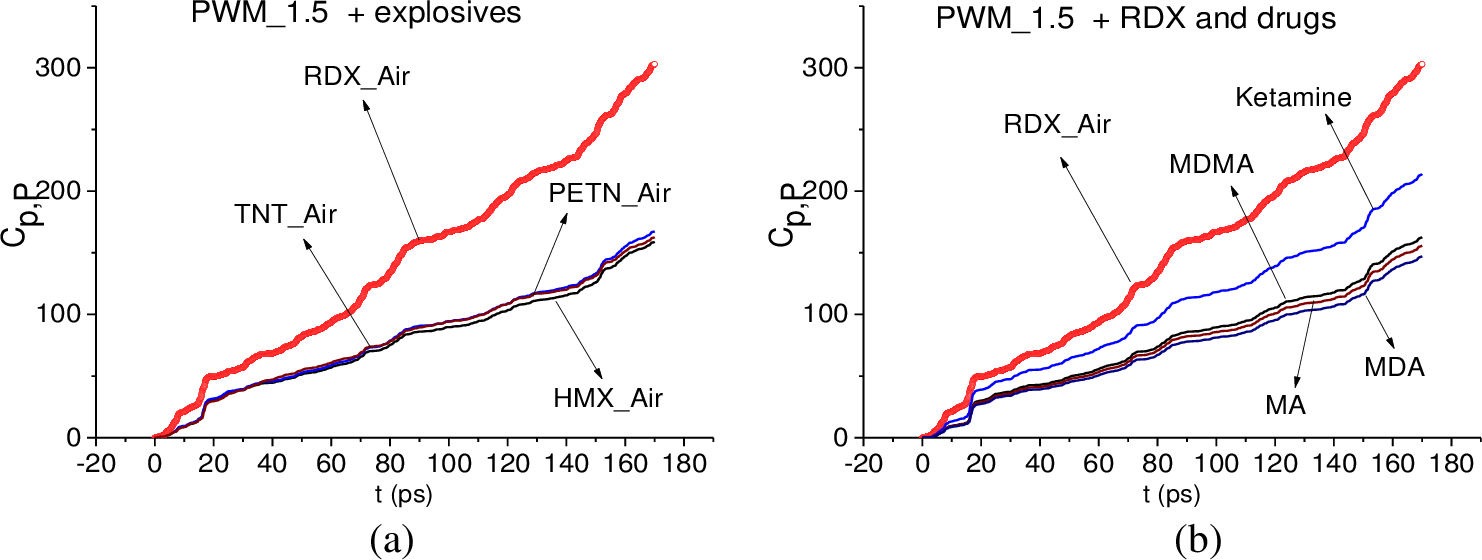
ICC *C*_*s*,*S*_ for the signal PWM_1.5 and the standard signals (a) RDX_Air, HMX_Air, PETN_Air, TNT_Air and (b) MA, MDA, MDMA, Ketamine in the time interval t = [0, 180] ps.

In Fig 19 the integral correlation criterion *C*_*s*,*S*_ (6) is shown for the signal PWM_1.5 and the standard signals RDX_Air, HMX_Air, PETN_Air, TNT_Air (a) and MA, MDA, MDMA, Ketamine signals (b) in the full time interval t = [0, 180] ps. In both cases, the integral correlation between the signal PWM_1.5 and the standard signal RDX_Air is greater than for other standard signals from explosives and drugs. The same results are valid in the short time intervals t = [0, 40] ps, [40, 70] ps, [140, 170] ps, which contain the main pulse, the first and the last sub-pulses of the signal PWM_1.5, correspondingly.

However, to confirm the presence of the substance RDX absorption frequencies in the signal PWM_1.5, it is necessary to analyze its spectral properties and use the ICC. We will do this in several time intervals.

#### 3.2.2. Analysis in the time interval t = [0, 25] ps, containing the main pulse

**Fig 20:**
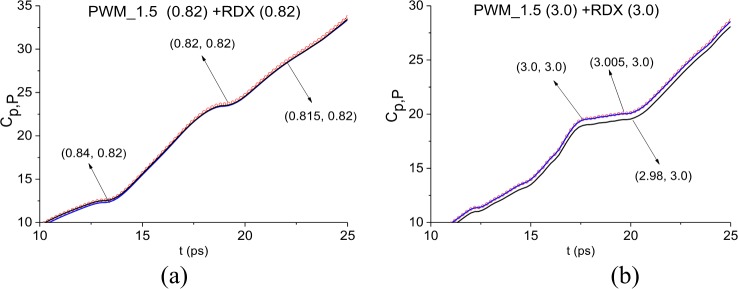
(a), (b) Time-dependent ICC *C*_*p*,*P*_ calculated for the frequencies (a) ν = 0.82 THz, (b) ν = 3.0 THz, in the frequency ranges *ν* = [0.815, 0.84] THz, [2.98, 3.005] THz, correspondingly.

Below in Fig 20, we use the ICC *C*_*p*,*P*_(*t*_*n*_) (a), (b) for detecting the absorption frequencies ν = 0.82 (a), 3.0 (b) THz in the main pulse of the signal PWM_1.5. For this purpose, we use the FDR’s of the minimal length, which in this case are: ν = [0.815, 0.84] THz for (a) and ν = [2.98, 3.005] THz for (b). The frequencies *ν* = 0.82, 3.0 THz are detected by ICC *C*_*p*,*P*_(*t*_*n*_) as the RDX absorption frequencies in the PWM_1.5 main pulse. The ICC *C*_*p*,*P*_(*t*_*n*_) evolution is shown in the shortened time interval t = [10, 25] ps in order to demonstrate the topmost position of the corresponding lines. Note that in the case of concave surface the minimal FDR’s are shorter than in the case of the rough surface (see Section 3.1.3, Figs 8 and 9). This result can be explained by the fact that the concave surface distorts the spectrum of the main pulse significantly stronger than the rough surface does. The ICC *CW*1_*p*,*P*_(*t*_*n*_) also demonstrates the detection of these frequencies (not shown).

The results obtained in Section 3.1.1 with the help of the ICC *C*_*s*,*S*_, confirm this conclusion. Nevertheless, in the next sections, we provide additional investigation in the time intervals, which do not contain the PWM_1.5 main pulse.

#### 3.2.3. Analysis in the time interval t = [40, 65] ps, containing the first sub-pulse

**Fig 21:**
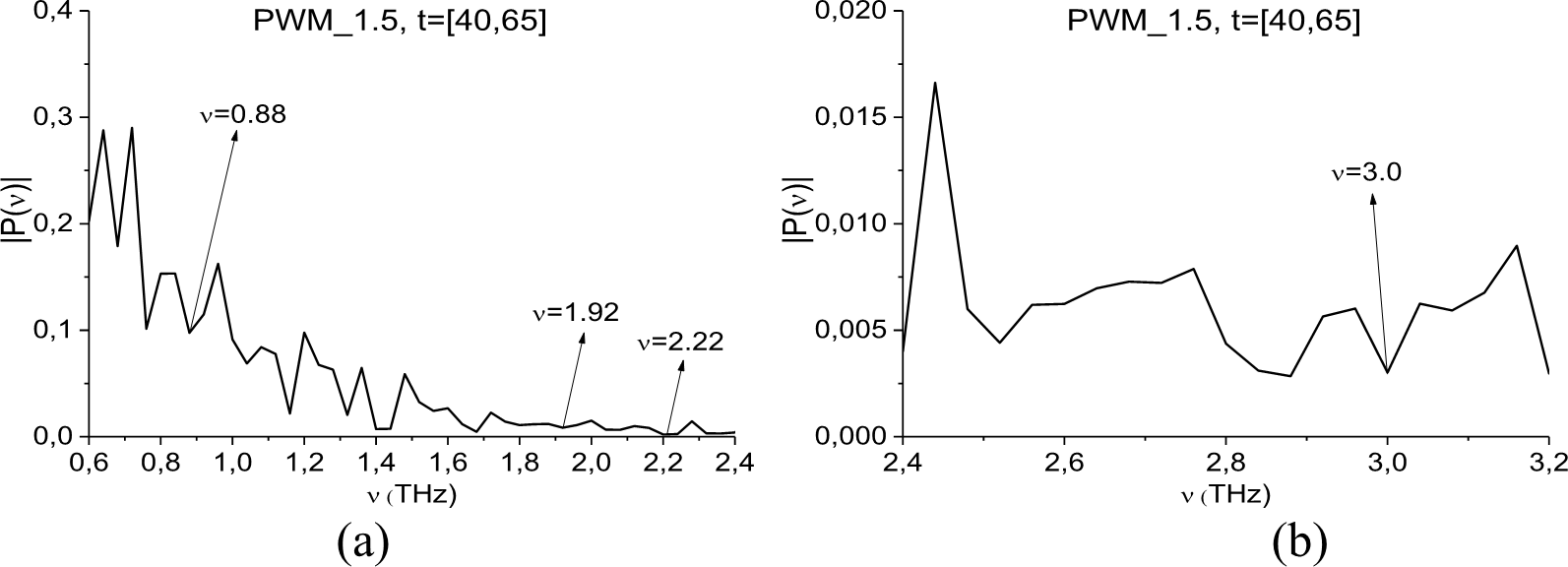
Fourier spectrum of the PWM_1.5 first sub-pulse in the frequency ranges (a) ν = [0.6, 2.4] THz, (b) [2.4, 3,2] THz. The spectral resolution is equal to ν = 0.04 THz.

As above, first we study the spectral properties of the PWM_1.5 signal in the time interval t *=* [40, 65] ps, which contains only the first sub-pulse. In Fig 21 the Fourier spectrum of the PWM_1.5 first sub-pulse is depicted in the frequency ranges ν = [0.6, 2.4] THz (a), [2.4, 3.2] THz (b). The first sub-pulse spectrum contains minima at the frequencies ν = 0.88, 1.92, 2.2, 3.0 THz close to the absorption frequencies of the transmitted RDX_Air signal at ν = 0.82, 1.92, 2.2, 3.0 THz, correspondingly. In the Reference signal spectrum (Fig 10) the minima at the frequencies ν = 0.88, 1.92, 2.2, 3.0 THz are absent, so environment influence is absent too. The corresponding spectral resolution is equal to ν = 0.04 THz. The spectral line dynamics of the transmitted RDX_Air signal at the frequencies ν = 0.82, 1.95, 2.2, 3.0 THz are used as the standard spectral line dynamics.

**Fig 22:**
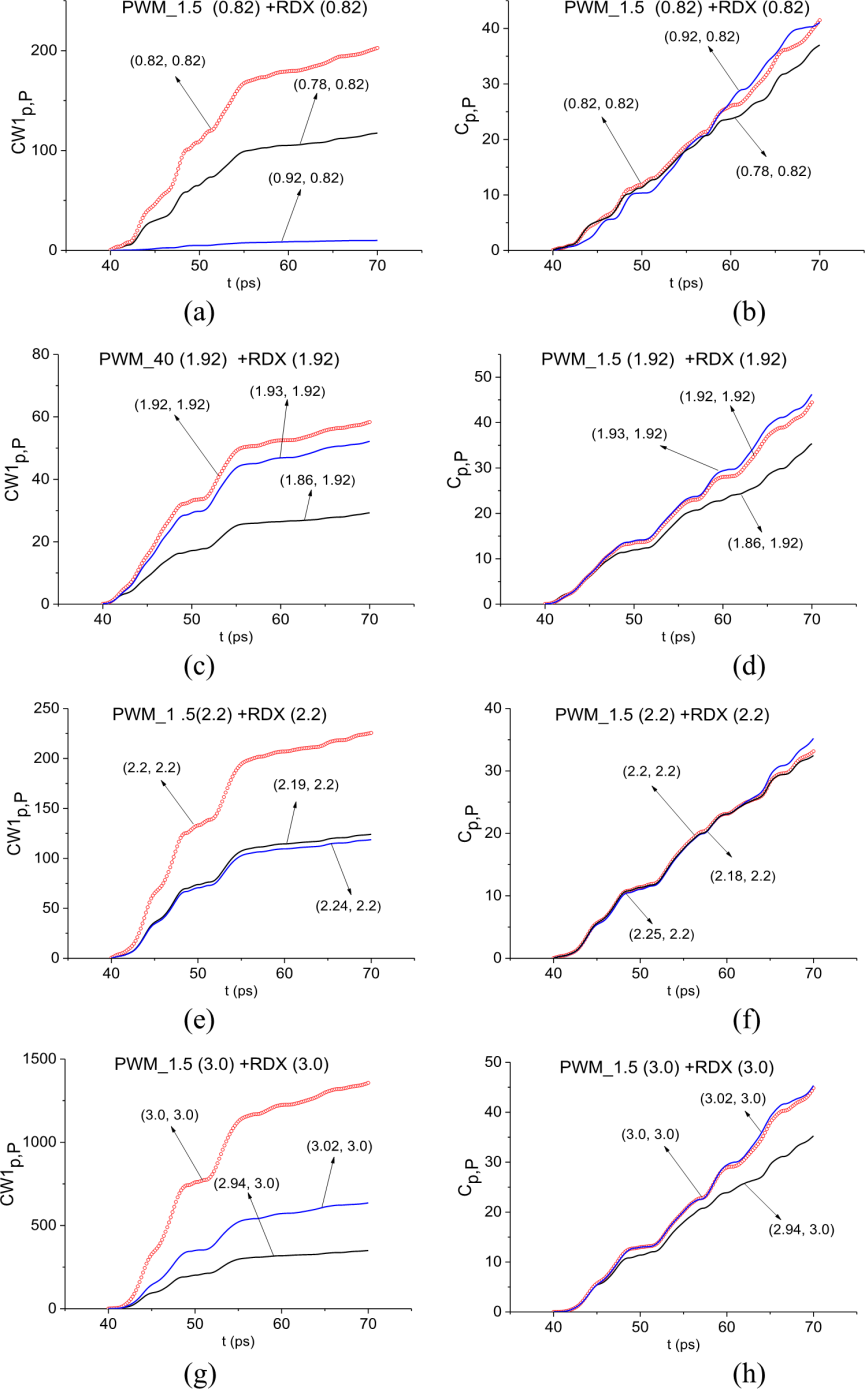
The ICC *CW*1_*p*,*P*_(*t*_*n*_) and *C*_*p*,*P*_(*t*_*n*_) evolution calculated for the frequencies (a), (b) ν = 0.82 THz, (c), (d) 1.92 THz, (e), (f) 2.2 THz and (g), (h) 3.0 THz in the time interval t = [40, 70] ps.

In Fig 22 the ICC *CW*1_*p*,*P*_ and *C*_*p*,*P*_ evolution is shown at the frequencies ν = 0.82 THz (a), (b), 1.92 THz (c), (d), 2.2 THz (e), (f) and 3.0 THz (g), (h). The corresponding minimal FDR’s are: *ν* = [0.78, 0.92] THz (a) (b), [1.86, 1.93] THz (c), (d), [2.19, 2.24] THz (e), (f), and [2.94., 3.02] THz (g), (h). In all cases, both ICC detect these frequencies as the absorption frequencies of RDX in the PWM_1.5 first sub-pulse. The time intervals, where the ICC detect the corresponding absorption frequency simultaneously, are t = [40, 57] ps (a), (b), [40, 48] ps (c), (d), [40, 58] ps (e), (f), [40, 48] ps (g), (h).

#### 3.2.4. Analysis in the time interval t = [70, 170] ps

In this section we briefly report about possibility to identify RDX absorption frequencies in the signal PWM_1.5 by means of the ICC’s in the remote time interval *t* = [70, 170 ] ps. It contains only the subsequent sub-pulses whose amplitudes are significantly less than the amplitude of the main pulse and the first sub-pulse. The analysis is similar to that for the PWM_40 signal.

The Fourier spectrum of the PWM_1.5 signal was calculated in the time interval *t* = [70, 170] ps with frequency resolution Δν = 0.01 THz. It contains minima at the frequencies ν = 0.84, 1.95, 2.19, 3.01 THz, which are close to absorption frequencies of RDX. The Reference spectrum minima (Fig 14) at these frequencies are absent, so the influence of environment is also absent.

**Fig 23:**
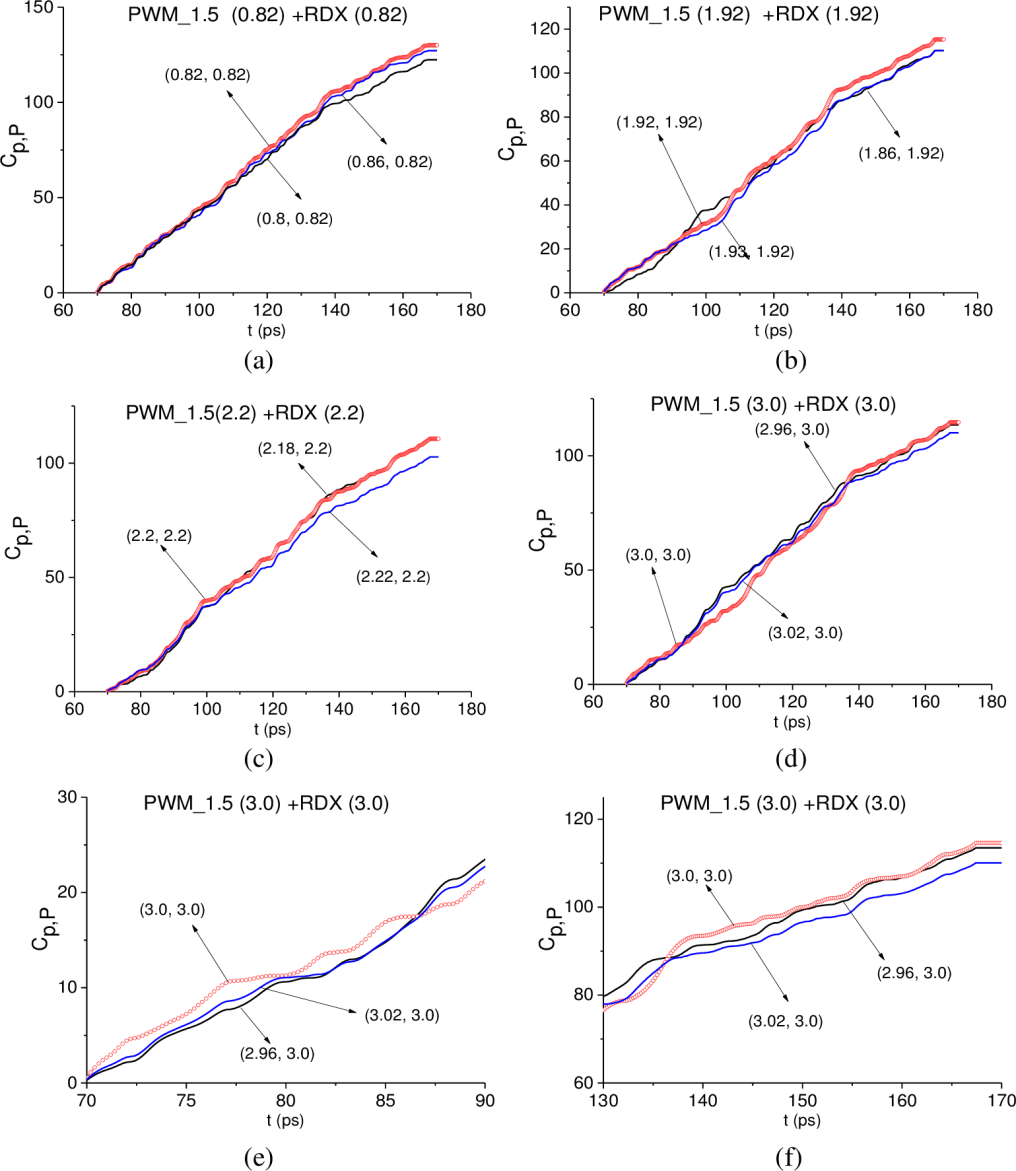
ICC *C*_*p*,*P*_(*t*_*n*_) detecting frequencies (a) ν = 0.82 THz, (b) 1.92 THz, (c) 2.2 THz and (d) 3.0 THz in the time interval t = [70, 170] ps; detecting the frequency ν = 3.0 THz in the time intervals (e) t = [70, 90] ps and (f) [130, 170] ps.

In all cases, the ICC *CW*1_*p*,*P*_ detects these frequencies as RDX absorption frequencies in the signal PWM_1.5 in the time interval t = [70, 170] ps (not shown). In Fig 23 the ICC *C*_*p*,*P*_ evolution is depicted at frequencies ν = 0.82 THz (a), 1.92 THz (b), 2.2 THz (c) and 3.0 THz (d). The corresponding minimal FDR’s are: *ν* = [0.8, 0.86] THz (a), [1.86, 1.93] THz (b), [2.18, 2.22] THz (c) and [2.96., 3.04] THz (d). Note that the ICC *C*_*p*,*P*_ detects frequencies ν = 0.82, 1.92, 3.0 THz in the shortened time intervals corresponding to the sub-pulse *S*_2_(*t*) (a), (b), (d) and *S*_3_(*t*) (d). The frequency ν = 2.2 THz is detected by the ICC *C*_*p*,*P*_ in the full time interval t = [70, 170] ps (c).

In (e), (f) the magnified view of Fig 23(D) is shown in the shortened time intervals t = [70, 90] ps (e) and t = [130, 170] ps (f) corresponding to the sub-pulses *S*_2_(*t*) and *S*_3_(*t*). One can see that the line corresponding to the frequency ν = 3.0 THz, is the topmost in these time intervals. Thus, this frequency is detected by the ICC *C*_*p*,*P*_ as the RDX absorption frequency.

## 4. Conclusions

We propose an effective time-dependent THz spectroscopy method, which allows detecting and identifying a substance with inhomogeneous surface using only one long-duration measured THz signal reflected from this sample without averaging of the measured THz signals over the viewing angles and scanning over the surface area. For successful analysis and identification, the registered THz signal must contain both the main reflected THz pulse and several sub-pulses. This feature of the method significantly increases the signal processing speed and allows us to use it in real time.

We showed that in contrast to a THz signal reflected from a smooth PWM surface, in the THz signals reflected from an inhomogeneous surface, the absorption frequencies of the substance are present both in the main pulses and sub-pulses. In particular, the main pulses of the THz signals reflected from a rough surface contain information about high absorption frequencies of the substance RDX. Thus, the high absorption frequencies are less distorted by inhomogeneity of the surface than lower frequencies and can be used for the detection and identification.

The identification is based on the SDA-method together with the integral correlation criteria. We compare the absorption spectral line dynamics of a substance under analysis with the corresponding dynamics for a standard substance from database. For the reliable and effective substance detection, we use several ICC’s simultaneously c with significantly less amplitude. This increases the reliability and accuracy of identification and decreases the probability of false positive and negative alarms appearing. It is worth noting that detection of RDX absorption frequencies in the sub-pulses with amplitude, which are significantly less than the amplitude of the main pulse, opens up the possibility of substance identification at long distances of about 3–10 meters, since in this case the noisy signal under analysis will not contain a pronounced THz pulse.

We use for identification those ICC’s which are less dependent on spectral characteristics of a signal under investigation and rely upon those of the standard signal from database only. This allows us to increase algorithm performance and decrease the influence of random fluctuations due to the rough or concave surface. In order to enhance the detection reliability we propose to use a few integral criteria simultaneously. As a result, one can detect not only the single substance absorption frequency with maximal spectral amplitude, which takes place at using the methods of averaging over the viewing angles and scanning over the surface area.

In conclusion, the SDA-method together with the discussed ICC’s is a promising and competitive tool for the effective detection and identification of substances with inhomogeneous surface. The discussed ICC’s demonstrate both high probability of the substance identification and a reliability of realization of the technique in practice. They can be very useful for the design of future THz security screening systems as well as for quality control in the pharmaceutical and food industry.
